# High‐throughput selective sweep SNP‐guided cloning of cold‐tolerance genes in rice

**DOI:** 10.1111/pbi.14329

**Published:** 2024-03-07

**Authors:** Xiaoxia Li, Hongmei Wang, Chujian Xiao, Juan Huang, Yuting Long, Chuxin Lin, Yue Zhu, Man Wang, Yao‐Guang Liu, Qunyu Zhang

**Affiliations:** ^1^ State Key Laboratory for Conservation and Utilization of Subtropical Agro‐Bioresources, Guangdong Laboratory for Lingnan Modern Agriculture, College of Life Sciences South China Agricultural University Guangzhou China

**Keywords:** QTL cloning, selective sweep SNP, cold tolerance, rice

The genetic dissection and cloning of quantitative trait loci (QTLs) are prerequisites for implementing genomics‐based applications in breeding programs. However, traditional positional cloning for QTLs is difficult, laborious and time‐consuming. For example, more than 250 and 600 QTLs for cold tolerance have been detected in rice using genetic mapping and genome‐wide association studies, respectively, but only 15 of these have been positionally cloned (Li *et al*., 2022).

We previously reported the identification of 5636 non‐synonymous SNPs in protein‐coding genes fixed in at least one rice subspecies, based on a genome‐wide selective sweep analysis of 655 *japonica* and 1205 *indica* accessions. These accessions were selected from 2673 landraces through SNP‐assisted principle component analysis, excluding 813 admixed accessions. We classified these selective sweep SNPs (SSNPs) into three types, *j*S (*japonica*‐selected), *i*S (*indica*‐selected) and *ji*S (*japonica*‐ and *indica*‐selected) (Wang *et al*., 2022). As the two subspecies differ markedly in their adaptation to different environmental temperatures, SSNPs could thus serve as markers assisting candidate‐gene selection for cloning cold‐tolerance QTL genes. To efficiently identify new cold‐tolerance genes, we built a comparative genomics analysis and QTL‐associated cloning platform, called SSNP‐guided cloning (SSNPC). As a case study for this method, we attempted to clone causal genes of 11 primarily mapped cold‐tolerance QTLs (Figure 1a).

**Figure 1:**
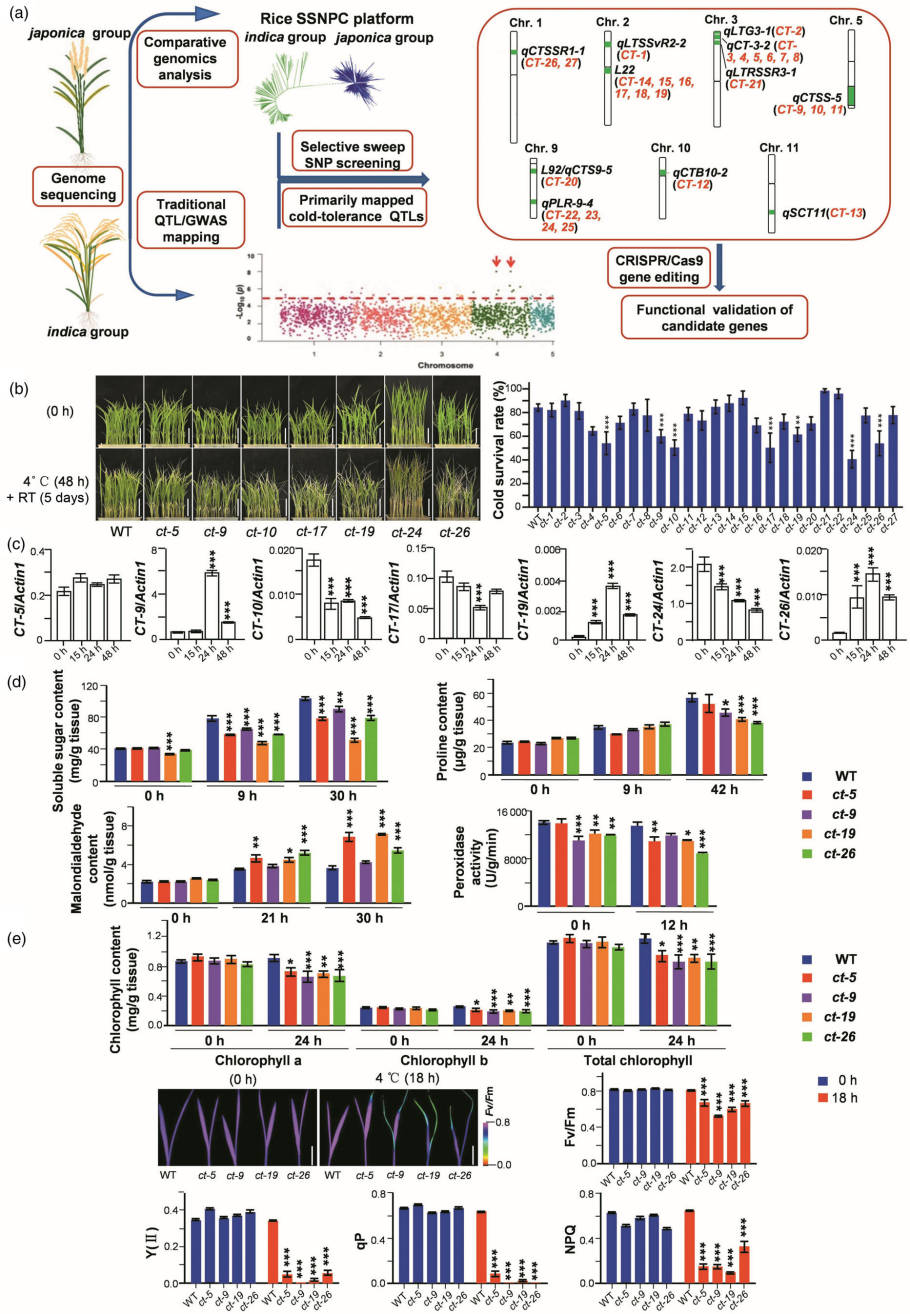
The selective sweep SNP‐guided cloning (SSNPC) method applied to cold‐tolerance QTLs. (a) Flow chart illustrating the use of SSNPC. The selected candidate genes (in red) were identified in 11 cold‐tolerance QTLs (in green). (b) Phenotypic response to cold stress in wild type (WT; Nipponbare) and the mutant lines each carrying a homozygous mutation of one of the 26 candidate genes, except for the male‐sterile *ct‐23*. The seven cold‐sensitive mutant lines are shown on the left. Scale bars = 5 cm. (c) Expression of the cold‐tolerance genes in WT treated at 4 °C for different durations. ***, significant difference between the cold treatments and the baseline (0 h) with *P* ≤ 0.001, *n* = 3. Changes in the cold‐stress‐related physiological indices observed in four cold‐sensitive mutants subjected to 4 °C are shown in (d and e). Scale bars = 2 cm. Two‐leaf‐stage seedlings were used. (b, d and e) *, ** and ***, significant differences between the mutants and WT with *P* < 0.05, *P* ≤ 0.01 and *P* ≤ 0.001, respectively, *n* = 3.

The 11 QTL regions (~2 Mbp each) encompass a total of 4588 predicted genes, 2901 of which have non‐synonymous SNPs between the *japonica* and *indica* groups. To detect signals of strong selection, we used a lab‐developed software package (RGVD, REAG and RCRAP) to calculate pooled heterozygosity (*H*_P_ = 2∑*n*_MAJ_∑*n*_MIN_/(∑*n*_MAJ_ + ∑*n*_MIN_)^2^, where *n*_MAJ_ and *n*_MIN_ are counts of the most and least abundant allele for each SNP, respectively; Axelsson *et al*., 2013) and fixation index (*F*_ST_) values in sliding 10‐kb windows for all these SNPs. In rice, we applied the cutoffs of 0.001 and 0.95 for *H*_P_ and *F*_ST_, respectively, to define selective sweeps with reduced *H*_P_ and/or increased *F*_ST_, which reflect strong selection during the evolution and domestication of *japonica* and *indica* rice populations. Thus, the SNPs within the selective sweeps, namely SSNPs, may assist in picking candidate genes with ‘large’ effects on cold tolerance. Indeed, using this algorithm we detected SSNPs in 11 of the 15 positionally cloned cold‐tolerance genes in rice (Table S1). We therefore selected genes containing one or more SSNPs of the *j*S and *ji*S types (*H*_P_‐*japonica* < 0.001 and/or *F*_ST_ > 0.95) as cold‐tolerance gene candidates in these 11 QTL regions. In this way, 27 candidate genes stood out from 2901 genes with non‐synonymous SNPs; these candidate genes were denoted in numerical order as *Cold Tolerance‐1* (*CT‐1*) to *CT‐27* (Figure 1a; Tables S2 and S3). The reduced *H*_P_ scores of divergent haplotypes of gene loci may also reflect the natural selection of the genes. We analysed the *H*_P_ scores for the haplotype SNPs in the coding regions of the 27 SSNP‐selected candidate genes and found that only three of them (*CT‐1*, *CT‐6* and *CT‐21*) had *H*_p‐_ *japonica* scores of <0.001, as compared to 18 genes with the SSNPs having these low *H*_p‐_ *japonica* scores (Tables S3 and S4). Therefore, SSNPs should be more suitable than haplotypes for candidate selection.

Using the plant CRISPR/Cas9 system (Ma *et al*., 2015; Tables S5 and S6), we knocked out each of these 27 candidate genes in a *japonica* variety, Nipponbare and obtained homozygous mutant lines (*ct‐1* to *ct‐27*, T_1_ and T_2_ generations, *ct‐23* was male‐sterile). We investigated their cold sensitivities by subjecting 15‐day‐old Nipponbare (WT) and mutant seedlings to 4 °C for 48 h, then returned them to room temperature (RT) for 5 days to allow growth recovery. Seven mutant lines, *ct‐5*, *ct‐9*, *ct*‐*10*, *ct‐17*, *ct‐19*, *ct‐24* and *ct‐26*, showed significantly decreased seedling survival rates compared to the WT (Figure 1b). Six of these seven *CT* genes (except *CT‐5*) were cold‐responsive at the mRNA level (Figure 1c; Table S7).

To evaluate cold tolerance in the mutant lines, we subjected four representative mutant lines (*ct‐5*, *ct‐9*, *ct‐19* and *ct‐26*) to 4 °C and measured the physiological indices including soluble sugars, proline, malondialdehyde (MDA), peroxidase (POD), chlorophyll and various chlorophyll fluorescence parameters (the maximal quantum yield of dark‐adapted leaves *F*_v_/*F*_m_, the effective quantum yield of illuminated leaves Y(II), photochemical quenching qP and nonphotochemical quenching NPQ). After the cold treatment, all mutants showed lower soluble sugar contents than WT plants and some also displayed lower proline amount, more MDA accumulations and lower POD activities than the WT (Figure 1d). All mutants also demonstrated significant decreases in chlorophyll *a*, chlorophyll *b*, total chlorophyll and chlorophyll fluorescence parameter values (Figure 1e), indicative of impaired photosynthesis. These observations confirmed the involvement of these mutants in cold signalling.

Our SSNPC method is more effective and enables higher throughput than traditional positional cloning of adaptive QTLs. It does not require the breeding of mapping populations and laborious genetic mapping to narrow down candidate intervals but uses SSNPs instead identified from genomics data of (sub)species/ecotypes for candidate selection. Our test study showed that this technique can identify and functionally validate multiple authentic cold‐tolerance genes in rice within a short timeframe (<2 years), as compared to many years spent in map‐based cloning of one single QTL. Our SSNPC could be leveraged into QTL cloning systems for other adaptive traits in rice and other crops. Our selective‐sweep analysis also identified numerous SSNPs fixed in gene promoter regions (Wang *et al*., 2022). Recent studies indicated that natural variations in the *HAN1* promoter (Mao *et al*., 2019) and codon repeat variation in *COLD11* (Li *et al*., 2023) confer cold tolerance in rice. Therefore, the combined use of SSNPs in coding regions, promoter regions and structural variations may further improve the efficiency of the SSNPC method.

## Conflict of interest

The authors declare no conflict of interest.

## Author contributions

X.L., H.W., C.X., J.H., Y.L., C.L. and Y.Z. performed research. X.L., H.W., Y.L. and Q.Z. wrote the manuscript. M.W., Y.L. and Q.Z. conceived and supervised the study. All authors read and approved the final manuscript.

## Supporting information


**Table S1** The selection state of the SNPs between japonica and indica genomes in the 15 positionally cloned cold‐tolerance genes.

**Table S2** The genomic regions and selected candidate genes in the 11 mapped cold‐tolerance QTLs.

**Table S3** The candidate genes and their *H*_p_ and *F*_ST_ information in the 11 cold‐tolerance QTLs.

**Table S4** The haplotypes in the coding regions of the 27 SSNP‐selected candidate genes.

**Table S5** The genomic mutations in the homozygous mutant lines for the candidate genes.

**Table S6** Primers for introducing the single guide RNAs (sgRNAs) for the CRISPR/Cas9 gene editing of the candidate genes.
**Table S7** Primers used for the qRT‐PCR analysis of the cold‐tolerance genes.
