# Supplementary material for: High‐throughput selective sweep SNP‐guided cloning of cold‐tolerance genes in rice
Source: Plant Biotechnol J. 2024 Mar 7;22(8):2104–6. doi: 10.1111/pbi.14329 (PMC11258967; doi:10.1111/pbi.14329)
Supplement: Supplementary file 1 — Table S1 The selection state of the SNPs between japonica and indica genomes in the 15 positionally cloned cold‐tolerance genes. [file PBI-22-2104-s006.pdf]

Supporting Table S1. The selection state of the SNPs between japonica and indica genomes in the 15 positionally cloned cold-tolerance genes

geneName	Selected	Chrom	Pos	GTMin(*indica*)	GTMax(*indica*)	*H* p(*indica*)	GTMin(*japonica*)	GTMax(*japonica*)	*H* p(*japonica*)	*F* st	Annotation
qPSR10	No	Chr10	18595702	2(G)	2408(A)	0.001658	133(A)	1177(G)	0.182438	0.8138	LOC_Os10g34840.1:(+),up-2855bp
qPSR10	No	Chr10	18595720	35(C)	2373(T)	0.028647	100(T)	1210(C)	0.141017	0.8295	LOC_Os10g34840.1:(+),up-2837bp
qPSR10	No	Chr10	18595769	32(T)	2374(C)	0.026246	101(C)	1207(T)	0.142509	0.8304	LOC_Os10g34840.1:(+),up-2788bp
qPSR10	No	Chr10	18595809	668(C)	1738(T)	0.401111	93(T)	1217(C)	0.131905	0.4427	LOC_Os10g34840.1:(+),up-2748bp
qPSR10	No	Chr10	18595823	664(C)	1736(T)	0.400244	91(T)	1219(C)	0.12928	0.4463	LOC_Os10g34840.1:(+),up-2734bp
qPSR10	No	Chr10	18596110	31(T)	2379(G)	0.025395	105(G)	1205(T)	0.147456	0.8262	LOC_Os10g34840.1:(+),up-2447bp
qPSR10	No	Chr10	18596470	658(G)	1752(A)	0.396968	96(A)	1212(G)	0.136015	0.4444	LOC_Os10g34840.1:(+),up-2087bp
qPSR10	No	Chr10	18596502	30(C)	2378(T)	0.024607	104(T)	1206(C)	0.146173	0.8283	LOC_Os10g34840.1:(+),up-2055bp
qPSR10	No	Chr10	18596519	10(T)	2396(G)	0.008278	104(G)	1206(T)	0.146173	0.8445	LOC_Os10g34840.1:(+),up-2038bp
qPSR10	No	Chr10	18596533	26(A)	2362(G)	0.021538	102(G)	1208(A)	0.1436	0.8339	LOC_Os10g34840.1:(+),up-2024bp
qPSR10	No	Chr10	18596535	26(A)	2364(G)	0.021521	102(G)	1208(A)	0.1436	0.834	LOC_Os10g34840.1:(+),up-2022bp
qPSR10	jiS	Chr10	18596679	0(N)	2266(G)	0	0(N)	1204(T)	0	1	LOC_Os10g34840.1:(+),up-1878bp
qPSR10	No	Chr10	18596936	8(T)	2244(C)	0.00708	97(C)	1195(T)	0.138882	0.8531	LOC_Os10g34840.1:(+),up-1621bp
qPSR10	No	Chr10	18597147	617(A)	1743(C)	0.386179	92(C)	1216(A)	0.130778	0.463	LOC_Os10g34840.1:(+),up-1410bp
qPSR10	jiS	Chr10	18597186	0(N)	2302(A)	0	14(A)	1200(G)	0.022798	0.9772	LOC_Os10g34840.1:(+),up-1371bp
qPSR10	No	Chr10	18597196	12(G)	2340(A)	0.010152	96(A)	1210(G)	0.136207	0.8528	LOC_Os10g34840.1:(+),up-1361bp
qPSR10	No	Chr10	18597216	12(T)	2324(G)	0.010221	100(G)	1208(T)	0.141215	0.8476	LOC_Os10g34840.1:(+),up-1341bp
qPSR10	No	Chr10	18597238	12(G)	2366(T)	0.010042	103(T)	1207(G)	0.144888	0.8441	LOC_Os10g34840.1:(+),up-1319bp
qPSR10	No	Chr10	18597325	16(C)	2334(T)	0.013524	103(T)	1205(C)	0.14509	0.8404	LOC_Os10g34840.1:(+),up-1232bp
qPSR10	No	Chr10	18597332	2(A)	2350(G)	0.001699	122(G)	1188(A)	0.168913	0.8277	LOC_Os10g34840.1:(+),up-1225bp
qPSR10	No	Chr10	18597369	12(T)	2364(C)	0.01005	104(C)	1206(T)	0.146173	0.8427	LOC_Os10g34840.1:(+),up-1188bp
qPSR10	No	Chr10	18597423	12(A)	2380(G)	0.009983	104(G)	1198(A)	0.146994	0.842	LOC_Os10g34840.1:(+),up-1134bp
qPSR10	No	Chr10	18597548	12(T)	2364(G)	0.01005	100(G)	1202(T)	0.141812	0.8472	LOC_Os10g34840.1:(+),up-1009bp
qPSR10	No	Chr10	18597596	14(T)	2378(C)	0.011637	103(C)	1195(T)	0.146112	0.8412	LOC_Os10g34840.1:(+),up-961bp
qPSR10	No	Chr10	18597672	14(G)	2384(A)	0.011608	103(A)	1205(G)	0.14509	0.8423	LOC_Os10g34840.1:(+),up-885bp
qPSR10	No	Chr10	18597675	12(T)	2386(C)	0.009958	103(C)	1203(T)	0.145294	0.8437	LOC_Os10g34840.1:(+),up-882bp
qPSR10	No	Chr10	18597687	14(C)	2384(T)	0.011608	103(T)	1205(C)	0.14509	0.8423	LOC_Os10g34840.1:(+),up-870bp
qPSR10	No	Chr10	18597718	2(A)	2404(G)	0.001661	113(G)	1195(A)	0.157856	0.8391	LOC_Os10g34840.1:(+),up-839bp
qPSR10	No	Chr10	18597729	14(G)	2388(A)	0.011589	101(A)	1207(G)	0.142509	0.8449	LOC_Os10g34840.1:(+),up-828bp
qPSR10	No	Chr10	18597738	10(T)	2388(C)	0.008306	101(C)	1207(T)	0.142509	0.8482	LOC_Os10g34840.1:(+),up-819bp
qPSR10	jS	Chr10	18597867	142(G)	1764(A)	0.137902	0(N)	1216(G)	0	0.8612	LOC_Os10g34840.1:(+),up-690bp
qPSR10	jiS	Chr10	18597868	0(N)	1904(C)	0	11(C)	1205(A)	0.017928	0.982	LOC_Os10g34840.1:(+),up-689bp
qPSR10	No	Chr10	18597903	10(T)	2382(C)	0.008326	100(C)	1200(T)	0.142012	0.8487	LOC_Os10g34840.1:(+),up-654bp
qPSR10	No	Chr10	18597930	14(G)	2388(A)	0.011589	92(A)	1206(G)	0.131709	0.8559	LOC_Os10g34840.1:(+),up-627bp
qPSR10	No	Chr10	18597931	12(C)	2390(T)	0.009942	93(T)	1205(C)	0.13303	0.8562	LOC_Os10g34840.1:(+),up-626bp
qPSR10	No	Chr10	18597938	629(G)	1769(A)	0.386999	10(A)	1212(G)	0.016233	0.5685	LOC_Os10g34840.1:(+),up-619bp
qPSR10	No	Chr10	18597962	12(T)	2392(A)	0.009934	101(A)	1207(T)	0.142509	0.8466	LOC_Os10g34840.1:(+),up-595bp
qPSR10	No	Chr10	18597965	651(T)	1755(A)	0.394727	98(A)	1212(T)	0.138425	0.4451	LOC_Os10g34840.1:(+),up-592bp
qPSR10	No	Chr10	18598070	12(T)	2398(G)	0.009909	102(G)	1208(T)	0.1436	0.8455	LOC_Os10g34840.1:(+),up-487bp
qPSR10	No	Chr10	18598115	2(A)	2406(C)	0.00166	112(C)	1198(A)	0.156373	0.8407	LOC_Os10g34840.1:(+),up-442bp
qPSR10	No	Chr10	18598116	12(T)	2394(C)	0.009925	100(C)	1210(T)	0.141017	0.8481	LOC_Os10g34840.1:(+),up-441bp
qPSR10	No	Chr10	18598156	12(T)	2390(A)	0.009942	98(A)	1204(T)	0.139207	0.8499	LOC_Os10g34840.1:(+),up-401bp
qPSR10	No	Chr10	18598173	655(G)	1745(A)	0.396866	92(A)	1214(G)	0.130963	0.4491	LOC_Os10g34840.1:(+),up-384bp
qPSR10	No	Chr10	18598191	12(T)	2388(C)	0.00995	96(C)	1208(T)	0.1364	0.8528	LOC_Os10g34840.1:(+),up-366bp

qPSR10	No	Chr10	18598197	14(G)	2384(T)	0.011608	98(T)	1208(G)	0.138815	0.8487	LOC_Os10g34840.1:(+),up-360bp
qPSR10	jS	Chr10	18598220	590(C)	1766(T)	0.375424	0(N)	1218(C)	0	0.5991	LOC_Os10g34840.1:(+),up-337bp
qPSR10	No	Chr10	18598230	647(C)	1757(T)	0.393402	90(T)	1218(C)	0.128146	0.4562	LOC_Os10g34840.1:(+),up-327bp
qPSR10	No	Chr10	18598245	10(A)	2392(G)	0.008292	100(G)	1208(A)	0.141215	0.8495	LOC_Os10g34840.1:(+),up-312bp
qPSR10	No	Chr10	18598249	10(G)	2392(A)	0.008292	101(A)	1207(G)	0.142509	0.8482	LOC_Os10g34840.1:(+),up-308bp
qPSR10	No	Chr10	18598341	12(C)	2398(T)	0.009909	102(T)	1206(C)	0.143801	0.8453	LOC_Os10g34840.1:(+),up-216bp
qPSR10	No	Chr10	18598349	69(C)	2339(T)	0.055667	102(T)	1206(C)	0.143801	0.7998	LOC_Os10g34840.1:(+),up-208bp
qPSR10	No	Chr10	18598377	651(T)	1759(A)	0.394314	99(A)	1209(T)	0.139919	0.4443	LOC_Os10g34840.1:(+),up-180bp
qPSR10	No	Chr10	18598503	12(G)	2396(C)	0.009917	101(C)	1207(G)	0.142509	0.8466	LOC_Os10g34840.1:(+),up-54bp
qPSR10	No	Chr10	18598826	4(T)	2376(C)	0.003356	106(C)	1190(T)	0.150201	0.8453	LOC_Os10g34840.1:(+),ATG->ACG,M->T,no-syn,exon-1
qPSR10	No	Chr10	18598921	12(G)	2322(A)	0.01023	98(A)	1176(G)	0.142012	0.8468	LOC_Os10g34840.1:(+),GGC->AGC,G->S,no-syn,exon-1
qPSR10	No	Chr10	18599102	659(A)	1737(G)	0.398788	98(G)	1206(A)	0.139011	0.4393	LOC_Os10g34840.1:(+),GAC->GGC,D->G,no-syn,exon-1
qPSR10	No	Chr10	18599343	2(A)	2408(G)	0.001658	117(G)	1193(A)	0.162672	0.8342	LOC_Os10g34840.1:(+),exon-1-utr-3'
qPSR10	No	Chr10	18599363	12(T)	2396(C)	0.009917	105(C)	1203(T)	0.147662	0.8414	LOC_Os10g34840.1:(+),exon-1-utr-3'
qPSR10	No	Chr10	18599410	12(G)	2394(T)	0.009925	103(T)	1205(G)	0.14509	0.844	LOC_Os10g34840.1:(+),exon-1-utr-3'
qPSR10	No	Chr10	18599419	12(C)	2390(T)	0.009942	103(T)	1203(C)	0.145294	0.8437	LOC_Os10g34840.1:(+),exon-1-utr-3'
HAN1	No	Chr11	16982617	2(A)	2408(G)	0.001658	53(G)	1257(A)	0.077642	0.9205	LOC_Os11g29290.1:(+),up-2973bp
HAN1	No	Chr11	16982743	2(T)	2406(C)	0.00166	54(C)	1256(T)	0.079044	0.9191	LOC_Os11g29290.1:(+),up-2847bp
HAN1	jiS	Chr11	16982999	0(N)	1960(T)	0	0(N)	1310(A)	0	1	LOC_Os11g29290.1:(+),up-2591bp
HAN1	No	Chr11	16983085	2(T)	2408(C)	0.001658	54(C)	1256(T)	0.079044	0.9191	LOC_Os11g29290.1:(+),up-2505bp
HAN1	iS	Chr11	16985083	0(N)	2408(C)	0	56(C)	1254(T)	0.081841	0.9179	LOC_Os11g29290.1:(+),up-507bp
HAN1	No	Chr11	16985094	30(T)	2376(C)	0.024627	50(C)	1260(T)	0.073422	0.9018	LOC_Os11g29290.1:(+),up-496bp
HAN1	No	Chr11	16985139	2(G)	2406(A)	0.00166	52(A)	1258(G)	0.076238	0.9219	LOC_Os11g29290.1:(+),up-451bp
HAN1	No	Chr11	16985143	2(G)	2406(A)	0.00166	52(A)	1256(G)	0.07635	0.9218	LOC_Os11g29290.1:(+),up-447bp
HAN1	No	Chr11	16985354	2(T)	2408(C)	0.001658	52(C)	1258(T)	0.076238	0.9219	LOC_Os11g29290.1:(+),up-236bp
HAN1	No	Chr11	16985465	2(C)	2406(G)	0.00166	54(G)	1256(C)	0.079044	0.9191	LOC_Os11g29290.1:(+),up-125bp
HAN1	No	Chr11	16986786	2(T)	2300(C)	0.001736	54(C)	1202(T)	0.08229	0.9157	LOC_Os11g29290.1:(+),CAT->CAC,H->H,syn,exon-1
HAN1	iS	Chr11	16986969	0(N)	1848(C)	0	32(C)	1134(A)	0.053382	0.9465	LOC_Os11g29290.1:(+),CCA->CCC,P->P,syn,exon-1
HAN1	iS	Chr11	16987158	0(N)	2138(G)	0	48(G)	1148(T)	0.077046	0.9227	LOC_Os11g29290.1:(+),GCT->GCG,A->A,syn,exon-1
COLD11	No	Chr11	23943129	878(T)	920(C)	0.499727	568(C)	708(T)	0.493981	0.0038	LOC_Os11g40150.1:(+),intron-1
COLD11	No	Chr11	23943406	988(G)	1184(A)	0.495928	578(A)	702(G)	0.495308	0.0081	LOC_Os11g40150.1:(+),intron-1
COLD11	No	Chr11	23943597	995(C)	1257(T)	0.493232	616(T)	692(C)	0.498312	0.007	LOC_Os11g40150.1:(+),intron-1
COLD11	No	Chr11	23945036	1083(C)	1303(T)	0.495749	622(T)	688(C)	0.498731	0.0045	LOC_Os11g40150.1:(+),AAC->AAT,N->N,syn,exon-6
COLD11	No	Chr11	23945380	1109(G)	1167(A)	0.499675	621(A)	687(G)	0.498727	0.0008	LOC_Os11g40150.1:(+),intron-6
COLD11	No	Chr11	23945482	1078(T)	1294(G)	0.495854	623(G)	685(T)	0.498877	0.0042	LOC_Os11g40150.1:(+),intron-6

OsGSTZ2	No	Chr12	5760202	905(C)	1505(T)	0.469009	1(T)	1309(C)	0.001526	0.4521	LOC_Os12g10730.2:(+),up-264bp
OsGSTZ2	No	Chr12	5760244	904(T)	1506(C)	0.468802	1(C)	1309(T)	0.001526	0.4526	LOC_Os12g10730.1:(+),exon-1-utr-5'
OsGSTZ2	No	Chr12	5760865	4(A)	2380(T)	0.00335	61(T)	1249(A)	0.088793	0.9076	LOC_Os12g10730.1:(+),intron-2 ;LOC_Os12g10730.2:(+),intron-1
OsGSTZ2	jS	Chr12	5761285	922(A)	1486(G)	0.472571	0(N)	1310(A)	0	0.4458	LOC_Os12g10730.1:(+),ACA->ACG,T->T,syn,exon-3 ;LOC_Os12g10730.2:(+),ACA->ACG,T->T,syn,exon-2
OsGSTZ2	jS	Chr12	5762220	918(A)	1492(G)	0.471637	0(N)	1310(A)	0	0.4478	LOC_Os12g10730.1:(+),ATA->GTA,I->V,no-syn,exon-6 ;LOC_Os12g10730.2:(+),ATA->GTA,I->V,no-syn,exon-5
OsGSTZ2	jS	Chr12	5762620	916(G)	1490(T)	0.471542	0(N)	1310(G)	0	0.4481	LOC_Os12g10730.1:(+),intron-7 ;LOC_Os12g10730.2:(+),intron-6
OsGSTZ2	No	Chr12	5762635	4(T)	2404(C)	0.003317	61(C)	1249(T)	0.088793	0.9076	LOC_Os12g10730.1:(+),intron-7 ;LOC_Os12g10730.2:(+),intron-6
OsGSTZ2	No	Chr12	5762731	4(A)	2400(G)	0.003322	60(G)	1248(A)	0.087535	0.9089	LOC_Os12g10730.1:(+),intron-7 ;LOC_Os12g10730.2:(+),intron-6
OsGSTZ2	No	Chr12	5763067	4(A)	2400(G)	0.003322	61(G)	1247(A)	0.088922	0.9075	LOC_Os12g10730.1:(+),intron-8 ;LOC_Os12g10730.2:(+),intron-7
OsGSTZ2	jS	Chr12	5763291	920(T)	1486(G)	0.47233	0(N)	1308(T)	0	0.4463	LOC_Os12g10730.1:(+),intron-9 ;LOC_Os12g10730.2:(+),intron-8
OsGSTZ2	No	Chr12	5763345	5(A)	2405(T)	0.004141	61(T)	1249(A)	0.088793	0.9068	LOC_Os12g10730.1:(+),AAT->ATT,N->I,no-syn,exon-10 ;LOC_Os12g10730.2:(+),AAT->ATT,N->I,no-syn,exon-9
LTG1	No	Chr2	24766997	59(A)	1981(C)	0.05617	324(C)	942(A)	0.380854	0.5388	LOC_Os02g40860.1:(+),exon-1-utr-5'
LTG1	No	Chr2	24767711	196(G)	2214(T)	0.149427	253(T)	1057(G)	0.311661	0.5327	LOC_Os02g40860.1:(+),intron-2
LTG1	No	Chr2	24767900	58(G)	2348(A)	0.047051	250(A)	1060(G)	0.30884	0.6336	LOC_Os02g40860.1:(+),intron-2
LTG1	No	Chr2	24769443	58(C)	2346(G)	0.047089	251(G)	1059(C)	0.309783	0.6325	LOC_Os02g40860.1:(+),intron-5
LTG1	No	Chr2	24769546	58(A)	2350(T)	0.047012	250(T)	1060(A)	0.30884	0.6336	LOC_Os02g40860.1:(+),intron-5
LTG1	No	Chr2	24771257	1126(A)	1278(G)	0.498001	189(G)	1121(A)	0.246919	0.1671	LOC_Os02g40860.1:(+),intron-10
LTG1	No	Chr2	24771281	58(T)	2352(C)	0.046974	251(C)	1057(T)	0.310144	0.6322	LOC_Os02g40860.1:(+),intron-10
LTG1	No	Chr2	24771435	57(G)	2351(A)	0.046222	339(A)	971(G)	0.383624	0.5446	LOC_Os02g40860.1:(+),intron-11
LTG1	No	Chr2	24771763	59(T)	2349(C)	0.047803	255(C)	1055(T)	0.313531	0.6275	LOC_Os02g40860.1:(+),intron-13
LTG1	jS	Chr2	24772469	1185(A)	1225(T)	0.499862	0(N)	1310(A)	0	0.3402	LOC_Os02g40860.1:(+),exon-14-utr-3'
LTG1	No	Chr2	24772471	1087(T)	1323(C)	0.495205	162(C)	1148(T)	0.216743	0.202	LOC_Os02g40860.1:(+),exon-14-utr-3'
LGS1	jS	Chr2	28863317	272(G)	2138(A)	0.20025	0(N)	1310(G)	0	0.797	LOC_Os02g47280.1:(-),exon-4-utr-3'
LGS1	jS	Chr2	28863430	269(T)	2141(A)	0.198319	0(N)	1310(T)	0	0.799	LOC_Os02g47280.1:(-),exon-4-utr-3'

LGS1	jS	Chr2	28863920	264(C)	2146(A)	0.195088	0(N)	1310(C)	0	0.8023	LOC_Os02g47280.1:(-),CTG->CTT,L->L,syn,exon-3;LOC_Os02g47280.2:(-),CTG->CTT,L->L,syn,exon-4
LGS1	No	Chr2	28864330	1037(T)	1373(C)	0.490281	1(C)	1309(T)	0.001526	0.3964	LOC_Os02g47280.1:(-),intron-2;LOC_Os02g47280.2:(-),intron-3
LGS1	jS	Chr2	28864815	265(C)	2141(T)	0.19602	0(N)	1310(C)	0	0.8014	LOC_Os02g47280.1:(-),intron-2;LOC_Os02g47280.2:(-),intron-3
LGS1	jS	Chr2	28865250	267(A)	2143(G)	0.197029	0(N)	1310(A)	0	0.8003	LOC_Os02g47280.1:(-),intron-2;LOC_Os02g47280.2:(-),intron-3
LGS1	jS	Chr2	28865303	265(T)	2145(A)	0.195735	0(N)	1310(T)	0	0.8017	LOC_Os02g47280.1:(-),intron-2;LOC_Os02g47280.2:(-),intron-3
LGS1	No	Chr2	28865357	271(G)	2139(A)	0.199607	1(A)	1309(G)	0.001526	0.7961	LOC_Os02g47280.1:(-),intron-2;LOC_Os02g47280.2:(-),intron-3
LGS1	No	Chr2	28866691	260(G)	2108(A)	0.195484	2(A)	1252(G)	0.003185	0.7988	LOC_Os02g47280.1:(-),GGC->GTT,G->V,no-syn,exon-1;LOC_Os02g47280.2:(-),GGC->GTT,G->V,no-syn,exon-2
LGS1	No	Chr2	28866692	259(C)	2105(A)	0.195113	2(A)	1252(C)	0.003185	0.7991	LOC_Os02g47280.1:(-),GGC->GTT,G->V,no-syn,exon-1;LOC_Os02g47280.2:(-),GGC->GTT,G->V,no-syn,exon-2
LGS1	jS	Chr2	28866907	482(C)	1922(T)	0.320599	0(N)	1296(C)	0	0.6656	LOC_Os02g47280.2:(-),GCG->ACG,A->T,no-syn,exon-1
LGS1	jS	Chr2	28867406	271(G)	2135(A)	0.199897	0(N)	1304(G)	0	0.7973	LOC_Os02g47280.1:(-),up-409bp
LGS1	jS	Chr2	28867439	254(G)	2110(A)	0.191801	0(N)	1292(G)	0	0.8057	LOC_Os02g47280.1:(-),up-442bp
LGS1	No	Chr2	28867462	236(T)	1704(G)	0.213702	2(G)	1246(T)	0.0032	0.7797	LOC_Os02g47280.1:(-),up-465bp
LGS1	jS	Chr2	28867684	818(G)	1574(T)	0.450055	0(N)	1308(G)	0	0.4899	LOC_Os02g47280.1:(-),up-687bp
LGS1	jS	Chr2	28867696	266(C)	2130(T)	0.197387	0(N)	1308(C)	0	0.7999	LOC_Os02g47280.1:(-),up-699bp
LGS1	No	Chr2	28867722	813(G)	1571(A)	0.449453	2(A)	1302(G)	0.003063	0.4881	LOC_Os02g47280.1:(-),up-725bp
LGS1	jS	Chr2	28867743	253(A)	2115(G)	0.190852	0(N)	1300(A)	0	0.8067	LOC_Os02g47280.1:(-),up-746bp
LGS1	jS	Chr2	28867751	797(A)	1555(G)	0.448068	0(N)	1302(A)	0	0.4934	LOC_Os02g47280.1:(-),up-754bp
LGS1	jS	Chr2	28867820	261(C)	2111(T)	0.195853	0(N)	1300(C)	0	0.8015	LOC_Os02g47280.1:(-),up-823bp
LGS1	No	Chr2	28867912	12(T)	2392(C)	0.009934	457(C)	853(T)	0.45431	0.473	LOC_Os02g47280.1:(-),up-915bp
LGS1	jS	Chr2	28868108	808(C)	1578(T)	0.447927	0(N)	1310(C)	0	0.4936	LOC_Os02g47280.1:(-),up-1111bp
LGS1	jS	Chr2	28868166	263(T)	2135(G)	0.195292	0(N)	1308(T)	0	0.8021	LOC_Os02g47280.1:(-),up-1169bp
LGS1	No	Chr2	28868465	814(C)	1564(A)	0.450264	2(A)	1304(C)	0.003058	0.4867	LOC_Os02g47280.1:(-),up-1468bp
LGS1	jS	Chr2	28868537	271(T)	2121(C)	0.200917	0(N)	1308(T)	0	0.7963	LOC_Os02g47280.1:(-),up-1540bp
LGS1	jS	Chr2	28868538	263(T)	2129(A)	0.195722	0(N)	1306(T)	0	0.8017	LOC_Os02g47280.1:(-),up-1541bp
LGS1	jS	Chr2	28868547	265(T)	2135(C)	0.19645	0(N)	1308(T)	0	0.8009	LOC_Os02g47280.1:(-),up-1550bp
LGS1	jS	Chr2	28868565	1036(T)	1362(A)	0.490759	0(N)	1308(T)	0	0.3961	LOC_Os02g47280.1:(-),up-1568bp
LGS1	jS	Chr2	28868591	1030(G)	1362(T)	0.490368	0(N)	1304(G)	0	0.3975	LOC_Os02g47280.1:(-),up-1594bp
LGS1	jS	Chr2	28868618	271(G)	2117(A)	0.201211	0(N)	1306(G)	0	0.7959	LOC_Os02g47280.1:(-),up-1621bp
LGS1	jS	Chr2	28868668	264(C)	2108(A)	0.197822	0(N)	1302(C)	0	0.7995	LOC_Os02g47280.1:(-),up-1671bp
LGS1	jS	Chr2	28868696	269(C)	2099(T)	0.201387	0(N)	1296(C)	0	0.7958	LOC_Os02g47280.1:(-),up-1699bp
LGS1	No	Chr2	28868707	1007(C)	1333(T)	0.490295	2(T)	1296(C)	0.003077	0.395	LOC_Os02g47280.1:(-),up-1710bp

LGS1	jS	Chr2	28868744	839(G)	1259(A)	0.479962	0(N)	1300(G)	0	0.4282	LOC_Os02g47280.1:(-),up-1747bp
LGS1	jS	Chr2	28868785	273(A)	2093(T)	0.204142	0(N)	1302(A)	0	0.7929	LOC_Os02g47280.1:(-),up-1788bp
LGS1	jS	Chr2	28869086	267(T)	2129(C)	0.198036	0(N)	1308(T)	0	0.7993	LOC_Os02g47280.1:(-),up-2089bp
LGS1	jS	Chr2	28869185	263(A)	2143(G)	0.194723	0(N)	1308(A)	0	0.8027	LOC_Os02g47280.1:(-),up-2188bp
LGS1	jS	Chr2	28869268	267(C)	2137(A)	0.197459	0(N)	1308(C)	0	0.7999	LOC_Os02g47280.1:(-),up-2271bp
LGS1	jS	Chr2	28869282	263(T)	2141(C)	0.194865	0(N)	1310(T)	0	0.8026	LOC_Os02g47280.1:(-),up-2285bp
LGS1	jS	Chr2	28869298	1034(G)	1366(T)	0.490432	0(N)	1310(G)	0	0.3973	LOC_Os02g47280.1:(-),up-2301bp
LGS1	jS	Chr2	28869407	271(T)	2135(G)	0.199897	0(N)	1310(T)	0	0.7973	LOC_Os02g47280.1:(-),up-2410bp
LGS1	jS	Chr2	28869578	975(C)	1433(T)	0.481912	0(N)	1310(C)	0	0.4231	LOC_Os02g47280.1:(-),up-2581bp
LGS1	jS	Chr2	28869584	1037(C)	1373(T)	0.490281	0(N)	1310(C)	0	0.3978	LOC_Os02g47280.1:(-),up-2587bp
LGS1	jS	Chr2	28869845	273(C)	2133(A)	0.201183	0(N)	1310(C)	0	0.796	LOC_Os02g47280.1:(-),up-2848bp
LGS1	jS	Chr2	28869899	274(G)	2134(A)	0.20168	0(N)	1306(G)	0	0.7955	LOC_Os02g47280.1:(-),up-2902bp
LGS1	jS	Chr2	28869925	267(T)	2139(C)	0.197315	0(N)	1308(T)	0	0.8	LOC_Os02g47280.1:(-),up-2928bp
qLTG3-1	jS	Chr3	217106	782(T)	1624(G)	0.438765	0(N)	1296(T)	0	0.509	LOC_Os03g01320.1:(+),up-2871bp
qLTG3-1	jS	Chr3	217406	357(A)	2049(G)	0.252725	0(N)	1290(A)	0	0.7413	LOC_Os03g01320.1:(+),up-2571bp
qLTG3-1	jS	Chr3	217480	356(C)	2050(A)	0.25214	0(N)	1290(C)	0	0.7419	LOC_Os03g01320.1:(+),up-2497bp
qLTG3-1	jS	Chr3	217585	358(A)	2028(T)	0.255059	0(N)	1282(A)	0	0.7388	LOC_Os03g01320.1:(+),up-2392bp
qLTG3-1	jS	Chr3	217878	326(T)	2034(C)	0.238108	0(N)	1248(T)	0	0.757	LOC_Os03g01320.1:(+),up-2099bp
qLTG3-1	No	Chr3	218160	356(G)	2030(A)	0.253884	1(A)	1283(G)	0.001556	0.7385	LOC_Os03g01320.1:(+),up-1817bp
qLTG3-1	No	Chr3	218227	350(G)	2034(A)	0.250517	1(A)	1289(G)	0.001549	0.7422	LOC_Os03g01320.1:(+),up-1750bp
qLTG3-1	jS	Chr3	218251	354(A)	2038(T)	0.252183	0(N)	1290(A)	0	0.7419	LOC_Os03g01320.1:(+),up-1726bp
qLTG3-1	jS	Chr3	218575	360(A)	2048(T)	0.254302	0(N)	1294(A)	0	0.7396	LOC_Os03g01320.1:(+),up-1402bp
qLTG3-1	jS	Chr3	218647	521(T)	1889(C)	0.338895	0(N)	1292(T)	0	0.6441	LOC_Os03g01320.1:(+),up-1330bp
qLTG3-1	jS	Chr3	218713	521(A)	1885(T)	0.339303	0(N)	1294(A)	0	0.6437	LOC_Os03g01320.1:(+),up-1264bp
qLTG3-1	No	Chr3	218752	356(T)	2048(C)	0.252314	2(C)	1296(T)	0.003077	0.7387	LOC_Os03g01320.1:(+),up-1225bp
qLTG3-1	No	Chr3	218778	352(A)	2050(G)	0.250138	2(G)	1296(A)	0.003077	0.7411	LOC_Os03g01320.1:(+),up-1199bp
qLTG3-1	No	Chr3	218815	357(A)	2049(T)	0.252725	2(T)	1292(A)	0.003086	0.7383	LOC_Os03g01320.1:(+),up-1162bp
qLTG3-1	No	Chr3	218955	356(A)	2046(G)	0.252487	2(G)	1296(A)	0.003077	0.7385	LOC_Os03g01320.1:(+),up-1022bp
qLTG3-1	jS	Chr3	219051	521(A)	1887(T)	0.339099	0(N)	1294(A)	0	0.6439	LOC_Os03g01320.1:(+),up-926bp
qLTG3-1	No	Chr3	219222	521(G)	1875(A)	0.340326	2(A)	1292(G)	0.003086	0.6394	LOC_Os03g01320.1:(+),up-755bp
qLTG3-1	jS	Chr3	219254	521(C)	1871(T)	0.340737	0(N)	1292(C)	0	0.6419	LOC_Os03g01320.1:(+),up-723bp
qLTG3-1	jS	Chr3	219393	507(C)	1831(G)	0.339654	0(N)	1280(C)	0	0.6432	LOC_Os03g01320.1:(+),up-584bp
qLTG3-1	jS	Chr3	219488	496(T)	1830(A)	0.335539	0(N)	1252(T)	0	0.6481	LOC_Os03g01320.1:(+),up-489bp
qLTG3-1	No	Chr3	219586	10(T)	2026(C)	0.009775	2(C)	1236(T)	0.003226	0.987	LOC_Os03g01320.1:(+),up-391bp
qLTG3-1	jS	Chr3	219608	498(G)	1856(A)	0.333599	0(N)	1226(G)	0	0.6504	LOC_Os03g01320.1:(+),up-369bp
qLTG3-1	jS	Chr3	219612	496(G)	1854(A)	0.333032	0(N)	1222(G)	0	0.6511	LOC_Os03g01320.1:(+),up-365bp
qLTG3-1	jS	Chr3	219647	270(G)	1804(A)	0.226471	0(N)	1164(G)	0	0.7694	LOC_Os03g01320.1:(+),up-330bp
qLTG3-1	jS	Chr3	219850	514(C)	1886(T)	0.336599	0(N)	1276(C)	0	0.6469	LOC_Os03g01320.1:(+),up-127bp
qLTG3-1	No	Chr3	219970	331(C)	2051(G)	0.239299	2(G)	1252(C)	0.003185	0.7526	LOC_Os03g01320.1:(+),up-7bp
qLTG3-1	jS	Chr3	219998	337(C)	2051(G)	0.242414	0(N)	1272(C)	0	0.7524	LOC_Os03g01320.1:(+),exon-1-utr-5'<br>;LOC_Os03g01320.2:(+),exon-1-utr-5'

qLTG3-1	No	Chr3	220026	339(T)	2043(A)	0.244126	4(A)	1250(T)	0.006359	0.7443	LOC_Os03g01320.1:(+),exon-1-utr-5' ;LOC_Os03g01320.2:(+),exon-1-utr-5'
qLTG3-1	No	Chr3	220250	330(T)	2034(G)	0.240215	2(G)	1262(T)	0.00316	0.7516	LOC_Os03g01320.1:(+),TCA->GGA,S->G,no-syn,exon-1 ;LOC_Os03g01320.2:(+),TCA->GGA,S->G,no-syn,exon-1
qLTG3-1	No	Chr3	220251	331(C)	2035(G)	0.240654	2(G)	1262(C)	0.00316	0.7512	LOC_Os03g01320.1:(+),TCA->GGA,S->G,no-syn,exon-1 ;LOC_Os03g01320.2:(+),TCA->GGA,S->G,no-syn,exon-1
CTB2	No	Chr4	1986022	132(C)	2270(T)	0.103868	235(T)	1075(C)	0.294418	0.5951	LOC_Os04g04254.1:(-),exon-14-utr-3' ;LOC_Os04g04254.2:(-),exon-15-utr-3'
CTB2	No	Chr4	1986407	171(T)	1851(A)	0.154835	364(A)	914(T)	0.407395	0.4138	LOC_Os04g04254.1:(-),intron-13 ;LOC_Os04g04254.2:(-),intron-14
CTB2	No	Chr4	1986674	133(C)	2257(T)	0.105104	231(T)	1075(C)	0.291182	0.5974	LOC_Os04g04254.1:(-),intron-13 ;LOC_Os04g04254.2:(-),intron-14
CTB2	iS	Chr4	1986828	0(N)	2400(A)	0	196(A)	534(T)	0.392809	0.5761	LOC_Os04g04254.1:(-),intron-13 ;LOC_Os04g04254.2:(-),intron-14
CTB2	No	Chr4	1987258	137(C)	2271(G)	0.107314	630(G)	678(C)	0.499327	0.2593	LOC_Os04g04254.1:(-),intron-13 ;LOC_Os04g04254.2:(-),intron-14
CTB2	No	Chr4	1988391	16(T)	2392(A)	0.013201	561(A)	747(T)	0.489889	0.3874	LOC_Os04g04254.1:(-),intron-11 ;LOC_Os04g04254.2:(-),intron-12
CTB2	No	Chr4	1988972	73(C)	2331(A)	0.058888	212(A)	1098(C)	0.271285	0.6637	LOC_Os04g04254.1:(-),intron-9 ;LOC_Os04g04254.2:(-),intron-10
CTB2	No	Chr4	1989221	41(C)	2311(A)	0.034256	140(A)	1152(C)	0.193235	0.7705	LOC_Os04g04254.1:(-),intron-9 ;LOC_Os04g04254.2:(-),intron-10
CTB2	No	Chr4	1989351	271(A)	2133(C)	0.200042	119(C)	1103(A)	0.175796	0.6238	LOC_Os04g04254.1:(-),intron-9 ;LOC_Os04g04254.2:(-),intron-10
CTB2	No	Chr4	1989520	2(A)	2336(T)	0.001709	238(T)	528(A)	0.428335	0.5237	LOC_Os04g04254.1:(-),intron-9 ;LOC_Os04g04254.2:(-),intron-10
CTB2	iS	Chr4	1989535	0(N)	2338(A)	0	241(A)	525(G)	0.43127	0.5207	LOC_Os04g04254.1:(-),intron-9 ;LOC_Os04g04254.2:(-),intron-10
CTB2	iS	Chr4	1989581	0(N)	2342(C)	0	306(C)	462(T)	0.47937	0.4295	LOC_Os04g04254.1:(-),intron-9 ;LOC_Os04g04254.2:(-),intron-10
CTB2	No	Chr4	1989781	96(C)	2308(T)	0.076678	243(T)	587(C)	0.414112	0.4751	LOC_Os04g04254.1:(-),intron-9 ;LOC_Os04g04254.2:(-),intron-10
CTB2	No	Chr4	1990390	453(G)	1955(T)	0.305465	208(T)	1102(G)	0.267136	0.4266	LOC_Os04g04254.1:(-),intron-8 ;LOC_Os04g04254.2:(-),intron-9
CTB2	No	Chr4	1991186	4(C)	2384(A)	0.003344	264(A)	612(C)	0.421092	0.5332	LOC_Os04g04254.1:(-),intron-8 ;LOC_Os04g04254.2:(-),intron-9
CTB2	iS	Chr4	1991907	1(A)	2409(T)	0.00083	507(T)	547(A)	0.49928	0.3491	LOC_Os04g04254.1:(-),intron-7 ;LOC_Os04g04254.2:(-),intron-8

CTB2	No	Chr4	1992166	1101(G)	1307(A)	0.496341	160(A)	1138(G)	0.216144	0.1976	LOC_Os04g04254.1:(-),intron-7 ;LOC_Os04g04254.2:(-),intron-8
CTB2	No	Chr4	1992200	858(G)	1542(T)	0.459388	71(T)	1171(G)	0.107796	0.3762	LOC_Os04g04254.1:(-),intron-7 ;LOC_Os04g04254.2:(-),intron-8
CTB2	No	Chr4	1992281	926(T)	1480(A)	0.473491	613(A)	683(T)	0.498541	0.0199	LOC_Os04g04254.1:(-),intron-7 ;LOC_Os04g04254.2:(-),intron-8
CTB2	No	Chr4	1993246	139(C)	2271(T)	0.1087	234(T)	1076(C)	0.293437	0.5915	LOC_Os04g04254.1:(-),intron-7 ;LOC_Os04g04254.2:(-),intron-8
CTB2	No	Chr4	1996147	965(C)	1445(A)	0.480166	96(A)	1214(C)	0.135824	0.3097	LOC_Os04g04254.1:(-),intron-2 ;LOC_Os04g04254.2:(-),intron-3
CTB2	No	Chr4	1996460	137(C)	2273(T)	0.10723	235(T)	1075(C)	0.294418	0.5919	LOC_Os04g04254.1:(-),intron-2 ;LOC_Os04g04254.2:(-),intron-3
CTB2	No	Chr4	1998046	121(G)	2257(A)	0.096588	231(A)	1077(G)	0.290832	0.606	LOC_Os04g04254.2:(-),up-110bp
CTB2	No	Chr4	1998059	113(C)	2253(T)	0.090958	229(T)	1075(C)	0.289547	0.6128	LOC_Os04g04254.2:(-),up-123bp
CTB2	No	Chr4	1998252	922(C)	1462(T)	0.474347	96(T)	1210(C)	0.136207	0.3225	LOC_Os04g04254.2:(-),up-316bp
CTB4a	No	Chr4	2031589	622(C)	1788(T)	0.38296	95(T)	1215(C)	0.13452	0.4636	LOC_Os04g04330.2:(+),up-2884bp
CTB4a	No	Chr4	2032027	620(G)	1790(C)	0.382156	97(C)	1213(G)	0.137126	0.4622	LOC_Os04g04330.2:(+),up-2446bp
CTB4a	No	Chr4	2032088	18(T)	2392(C)	0.014826	334(C)	974(T)	0.380294	0.5786	LOC_Os04g04330.2:(+),up-2385bp
CTB4a	No	Chr4	2032103	619(A)	1791(T)	0.381753	99(T)	1211(A)	0.139723	0.4603	LOC_Os04g04330.2:(+),up-2370bp
CTB4a	No	Chr4	2032104	614(C)	1796(T)	0.379726	99(T)	1211(C)	0.139723	0.4629	LOC_Os04g04330.2:(+),up-2369bp
CTB4a	No	Chr4	2032277	623(G)	1787(A)	0.383362	98(A)	1212(G)	0.138425	0.4595	LOC_Os04g04330.2:(+),up-2196bp
CTB4a	No	Chr4	2032342	672(C)	1738(T)	0.402175	35(T)	1275(C)	0.052007	0.5146	LOC_Os04g04330.2:(+),up-2131bp
CTB4a	No	Chr4	2032368	17(A)	2393(G)	0.014008	334(G)	976(A)	0.379913	0.5799	LOC_Os04g04330.2:(+),up-2105bp
CTB4a	No	Chr4	2032577	549(A)	1857(G)	0.352227	149(G)	1161(A)	0.201607	0.4383	LOC_Os04g04330.2:(+),up-1896bp
CTB4a	No	Chr4	2032605	595(C)	1813(T)	0.372076	97(T)	1213(C)	0.137126	0.4746	LOC_Os04g04330.2:(+),up-1868bp
CTB4a	No	Chr4	2032629	552(A)	1858(G)	0.353167	105(G)	1167(A)	0.151466	0.4838	LOC_Os04g04330.2:(+),up-1844bp
CTB4a	No	Chr4	2032783	548(T)	1862(C)	0.351363	151(C)	1159(T)	0.203961	0.4371	LOC_Os04g04330.2:(+),up-1690bp
CTB4a	No	Chr4	2032946	667(C)	1743(T)	0.400331	34(T)	1274(C)	0.050636	0.5183	LOC_Os04g04330.2:(+),up-1527bp
CTB4a	No	Chr4	2033016	674(C)	1736(T)	0.402908	36(T)	1274(C)	0.053451	0.5122	LOC_Os04g04330.2:(+),up-1457bp
CTB4a	jiS	Chr4	2033332	0(N)	2186(T)	0	0(N)	1306(A)	0	1	LOC_Os04g04330.2:(+),up-1141bp
CTB4a	No	Chr4	2033352	72(G)	2332(C)	0.058106	46(C)	1264(G)	0.067763	0.874	LOC_Os04g04330.2:(+),up-1121bp
CTB4a	No	Chr4	2033466	140(G)	2266(T)	0.109604	45(T)	1265(G)	0.066342	0.8238	LOC_Os04g04330.2:(+),up-1007bp
CTB4a	No	Chr4	2033973	88(C)	2108(T)	0.076934	171(T)	1139(C)	0.22699	0.6933	LOC_Os04g04330.2:(+),up-500bp
CTB4a	No	Chr4	2034083	82(G)	2216(A)	0.06882	347(A)	963(G)	0.389442	0.5159	LOC_Os04g04330.2:(+),up-390bp
CTB4a	No	Chr4	2034538	16(T)	2394(C)	0.01319	402(C)	908(T)	0.425402	0.5175	LOC_Os04g04330.1:(+),exon-1-utr-5' ;LOC_Os04g04330.2:(+),exon-1-utr-5'
CTB4a	jiS	Chr4	2034634	0(N)	2112(T)	0	0(N)	1290(G)	0	1	LOC_Os04g04330.1:(+),exon-1-utr-5' ;LOC_Os04g04330.2:(+),exon-1-utr-5'
CTB4a	jiS	Chr4	2034637	0(N)	2220(T)	0	0(N)	1292(A)	0	1	LOC_Os04g04330.1:(+),exon-1-utr-5' ;LOC_Os04g04330.2:(+),exon-1-utr-5'

CTB4a	jiS	Chr4	2034638	0(N)	2408(C)	0	0(N)	1292(G)	0	1	LOC_Os04g04330.1:(+),exon-1-utr-5' ;LOC_Os04g04330.2:(+),exon-1-utr-5'
CTB4a	No	Chr4	2035097	532(G)	1830(C)	0.349006	221(C)	1061(G)	0.285339	0.3634	LOC_Os04g04330.1:(+),GCC->CCC,A->P,no-syn,exon-1 ;LOC_Os04g04330.2:(+),GCC->CCC,A->P,no-syn,exon-1
CTB4a	No	Chr4	2036614	538(C)	1854(G)	0.348658	218(G)	1082(C)	0.279143	0.3696	LOC_Os04g04330.1:(+),CTC->CTG,L->L,syn,exon-1 ;LOC_Os04g04330.2:(+),CTC->CTG,L->L,syn,exon-1
CTB4a	No	Chr4	2037043	51(A)	2343(C)	0.041699	516(C)	780(A)	0.479252	0.3923	LOC_Os04g04330.1:(+),CTA->CTC,L->L,syn,exon-1 ;LOC_Os04g04330.2:(+),CTA->CTC,L->L,syn,exon-1
CTB4a	No	Chr4	2037796	616(C)	1794(T)	0.380539	622(T)	676(C)	0.499135	0.0734	LOC_Os04g04330.1:(+),intron-1 ;LOC_Os04g04330.2:(+),intron-1
CTB4a	No	Chr4	2037848	574(T)	1836(G)	0.362895	627(G)	675(T)	0.49932	0.0829	LOC_Os04g04330.1:(+),intron-1 ;LOC_Os04g04330.2:(+),intron-1
CTB4a	No	Chr4	2038140	134(G)	2266(A)	0.105432	195(A)	1011(G)	0.271095	0.6188	LOC_Os04g04330.1:(+),GCC->ACC,A->T,no-syn,exon-2 ;LOC_Os04g04330.2:(+),GCC->ACC,A->T,no-syn,exon-2
COLD1	No	Chr4	30312542	715(C)	1695(T)	0.417322	39(T)	1271(C)	0.057769	0.488	LOC_Os04g51180.1:(-),intron-11 ;LOC_Os04g51180.2:(-),intron-8
COLD1	No	Chr4	30313719	711(T)	1695(C)	0.416369	41(C)	1269(T)	0.060636	0.4868	LOC_Os04g51180.1:(-),intron-7 ;LOC_Os04g51180.2:(-),intron-7
COLD1	No	Chr4	30314011	718(C)	1692(T)	0.418332	40(T)	1270(C)	0.059204	0.4852	LOC_Os04g51180.1:(-),intron-6 ;LOC_Os04g51180.2:(-),intron-6
COLD1	No	Chr4	30316102	688(C)	1622(A)	0.418259	44(A)	1216(C)	0.067402	0.4778	LOC_Os04g51180.1:(-),TCG->TCT,S->S,syn,exon-1 ;LOC_Os04g51180.2:(-),TCG->TCT,S->S,syn,exon-1
COLD1	No	Chr4	30316708	713(A)	1697(T)	0.416646	325(T)	985(A)	0.373084	0.2079	LOC_Os04g51180.2:(-),up-493bp
COLD1	No	Chr4	30316924	692(G)	1716(T)	0.409582	327(T)	977(G)	0.375766	0.213	LOC_Os04g51180.2:(-),up-709bp
COLD1	No	Chr4	30317459	2(T)	2408(C)	0.001658	328(C)	982(T)	0.375381	0.5975	LOC_Os04g51180.2:(-),up-1244bp
Ctb1	No	Chr4	31456073	77(G)	2333(C)	0.061859	506(C)	804(G)	0.474126	0.3866	LOC_Os04g52830.2:(+),up-2871bp
Ctb1	No	Chr4	31456163	201(G)	2209(A)	0.152893	206(A)	1104(G)	0.265047	0.5794	LOC_Os04g52830.2:(+),up-2781bp
Ctb1	No	Chr4	31456282	501(T)	1909(C)	0.329336	2(C)	1308(T)	0.003049	0.6525	LOC_Os04g52830.2:(+),up-2662bp
Ctb1	No	Chr4	31456862	888(A)	1466(G)	0.469855	295(G)	1009(A)	0.350097	0.1603	LOC_Os04g52830.2:(+),up-2082bp
Ctb1	No	Chr4	31456879	774(G)	1544(A)	0.444827	2(A)	1302(G)	0.003063	0.496	LOC_Os04g52830.2:(+),up-2065bp
Ctb1	No	Chr4	31456912	524(A)	1692(G)	0.361096	2(G)	1306(A)	0.003053	0.6142	LOC_Os04g52830.2:(+),up-2032bp
Ctb1	No	Chr4	31457893	355(C)	2049(T)	0.251728	5(T)	1303(C)	0.007616	0.7349	LOC_Os04g52830.2:(+),up-1051bp
Ctb1	No	Chr4	31457952	190(G)	2216(A)	0.145466	607(A)	703(G)	0.497315	0.2452	LOC_Os04g52830.2:(+),up-992bp
Ctb1	No	Chr4	31458127	632(T)	1210(C)	0.450768	2(C)	1302(T)	0.003063	0.4857	LOC_Os04g52830.2:(+),up-817bp

Ctb1	jiS	Chr4	31458602	46(A)	2112(T)	0.041723	0(N)	1230(A)	0	0.9582	LOC_Os04g52830.2:(+),up-342bp
Ctb1	No	Chr4	31458922	304(G)	1866(A)	0.240933	16(A)	1278(G)	0.024424	0.73	LOC_Os04g52830.2:(+),up-22bp
Ctb1	No	Chr4	31459020	274(T)	2024(C)	0.210035	22(C)	1214(T)	0.034965	0.7522	LOC_Os04g52830.1:(+),exon-1-utr-5' ;LOC_Os04g52830.2:(+),exon-1-utr-5'
Ctb1	jiS	Chr4	31459102	0(N)	2394(T)	0	0(N)	1076(A)	0	1	LOC_Os04g52830.2:(+),exon-1-utr-5'
Ctb1	No	Chr4	31459362	178(C)	2154(T)	0.141006	342(T)	712(C)	0.438384	0.382	LOC_Os04g52830.1:(+),intron-2 ;LOC_Os04g52830.2:(+),intron-1
Ctb1	No	Chr4	31459521	174(T)	2228(G)	0.134384	619(G)	691(T)	0.49849	0.246	LOC_Os04g52830.1:(+),intron-2 ;LOC_Os04g52830.2:(+),intron-1
Ctb1	No	Chr4	31460219	865(T)	1545(A)	0.460194	2(A)	1308(T)	0.003049	0.4685	LOC_Os04g52830.1:(+),intron-2 ;LOC_Os04g52830.2:(+),intron-1
Ctb1	No	Chr4	31460860	1167(G)	1243(C)	0.499503	2(C)	1308(G)	0.003049	0.3443	LOC_Os04g52830.1:(+),CTG->CTC,L->L,syn,exon-3 ;LOC_Os04g52830.2:(+),CTG->CTC,L->L,syn,exon-2
Ctb1	No	Chr4	31461280	310(T)	2100(C)	0.22417	22(C)	1288(T)	0.033024	0.7393	LOC_Os04g52830.1:(+),TCT->TCC,S->S,syn,exon-3 ;LOC_Os04g52830.2:(+),TCT->TCC,S->S,syn,exon-2
qCT7	jS	Chr7	16144962	681(G)	1717(C)	0.406676	0(N)	1310(G)	0	0.5572	LOC_Os07g27670.1:(+),up-2951bp
qCT7	jS	Chr7	16145038	684(G)	1684(A)	0.410832	0(N)	1310(G)	0	0.5514	LOC_Os07g27670.1:(+),up-2875bp
qCT7	No	Chr7	16145162	10(A)	2334(T)	0.008496	17(T)	1283(A)	0.025812	0.9657	LOC_Os07g27670.1:(+),up-2751bp
qCT7	No	Chr7	16148667	8(A)	2402(T)	0.006617	14(T)	1296(A)	0.021146	0.9722	LOC_Os07g27670.1:(+),CGA->CGT,R->R,syn,exon-2
qCT7	jS	Chr7	16148708	648(C)	1762(T)	0.393167	0(N)	1310(C)	0	0.5758	LOC_Os07g27670.1:(+),CCC->CTC,P->L,no-syn,exon-2
qCT7	jS	Chr7	16149147	805(C)	1593(T)	0.446009	0(N)	1300(C)	0	0.4969	LOC_Os07g27670.1:(+),GCT->GTT,A->V,no-syn,exon-3
qCT7	No	Chr7	16149449	9(A)	2401(G)	0.007441	17(G)	1293(A)	0.025617	0.9669	LOC_Os07g27670.1:(+),AAC->GAC,N->D,no-syn,exon-3
COLDF	No	Chr8	22687968	6(G)	2404(A)	0.004967	4(A)	1306(G)	0.006088	0.9889	LOC_Os08g36000.1:(-),GTC->GTT,V->V,syn,exon-3
COLDF	No	Chr8	22688814	6(A)	2400(G)	0.004975	2(G)	1306(A)	0.003053	0.992	LOC_Os08g36000.1:(-),GTC->GCC,V->A,no-syn,exon-1
COLDF	No	Chr8	22689098	6(A)	2404(G)	0.004967	4(G)	1306(A)	0.006088	0.9889	LOC_Os08g36000.1:(-),GGT->GGC,G->G,syn,exon-1
COLDF	No	Chr8	22689368	6(A)	2398(G)	0.004979	2(G)	1300(A)	0.003067	0.9919	LOC_Os08g36000.1:(-),GTT->GTC,V->V,syn,exon-1
COLDF	No	Chr8	22689418	6(C)	2396(A)	0.004983	2(A)	1304(C)	0.003058	0.9919	LOC_Os08g36000.1:(-),GTC->TTC,V->F,no-syn,exon-1
COLDF	jiS	Chr8	22689694	0(N)	2302(A)	0	15(A)	1293(C)	0.022673	0.9773	LOC_Os08g36000.1:(-),up-159bp
COLDF	No	Chr8	22689840	6(A)	2384(C)	0.005008	3(C)	1295(A)	0.004612	0.9904	LOC_Os08g36000.1:(-),up-305bp
COLDF	No	Chr8	22689858	6(T)	2376(G)	0.005025	3(G)	1293(T)	0.004619	0.9903	LOC_Os08g36000.1:(-),up-323bp

COLDF	No	Chr8	22689889	8(A)	2352(G)	0.006757	4(G)	1296(A)	0.006135	0.9871 LOC_Os08g36000.1:(-),up-354bp
COLDF	No	Chr8	22690050	4(C)	2220(T)	0.003591	2(T)	1268(C)	0.003145	0.9933 LOC_Os08g36000.1:(-),up-515bp
COLDF	No	Chr8	22690277	5(A)	2141(G)	0.004649	8(G)	1188(A)	0.013288	0.982 LOC_Os08g36000.1:(-),up-742bp
COLDF	No	Chr8	22690348	12(G)	2144(A)	0.01107	3(A)	1221(G)	0.00489	0.984 LOC_Os08g36000.1:(-),up-813bp
COLDF	No	Chr8	22690409	8(C)	2252(A)	0.007055	3(A)	1237(C)	0.004827	0.9881 LOC_Os08g36000.1:(-),up-874bp
COLDF	No	Chr8	22690591	6(G)	2386(A)	0.005004	3(A)	1303(G)	0.004584	0.9904 LOC_Os08g36000.1:(-),up-1056bp
COLDF	No	Chr8	22690618	6(C)	2386(T)	0.005004	3(T)	1307(C)	0.00457	0.9904 LOC_Os08g36000.1:(-),up-1083bp
COLDF	No	Chr8	22690622	6(G)	2386(A)	0.005004	3(A)	1307(G)	0.00457	0.9904 LOC_Os08g36000.1:(-),up-1087bp
COLDF	No	Chr8	22690660	6(C)	2374(T)	0.005029	3(T)	1305(C)	0.004577	0.9904 LOC_Os08g36000.1:(-),up-1125bp
COLDF	jiS	Chr8	22690810	0(N)	2376(T)	0	2(T)	1308(G)	0.003049	0.9969 LOC_Os08g36000.1:(-),up-1275bp
COLDF	No	Chr8	22690869	6(A)	2396(G)	0.004983	4(G)	1302(A)	0.006107	0.9889 LOC_Os08g36000.1:(-),up-1334bp
COLDF	No	Chr8	22691038	6(A)	2402(T)	0.004971	2(T)	1304(A)	0.003058	0.992 LOC_Os08g36000.1:(-),up-1503bp
COLDF	No	Chr8	22691042	6(C)	2402(T)	0.004971	2(T)	1304(C)	0.003058	0.992 LOC_Os08g36000.1:(-),up-1507bp
COLDF	No	Chr8	22691102	6(T)	2394(C)	0.004987	2(C)	1306(T)	0.003053	0.9919 LOC_Os08g36000.1:(-),up-1567bp
COLDF	No	Chr8	22691110	6(G)	2394(A)	0.004987	2(A)	1308(G)	0.003049	0.992 LOC_Os08g36000.1:(-),up-1575bp
COLDF	No	Chr8	22691126	6(A)	2392(G)	0.004992	3(G)	1305(A)	0.004577	0.9904 LOC_Os08g36000.1:(-),up-1591bp
COLDF	No	Chr8	22691214	6(A)	2396(G)	0.004983	3(G)	1305(A)	0.004577	0.9904 LOC_Os08g36000.1:(-),up-1679bp
COLDF	No	Chr8	22691226	6(C)	2398(A)	0.004979	3(A)	1297(C)	0.004605	0.9904 LOC_Os08g36000.1:(-),up-1691bp
COLDF	No	Chr8	22691453	6(G)	2366(C)	0.005046	2(C)	1302(G)	0.003063	0.9919 LOC_Os08g36000.1:(-),up-1918bp
COLDF	No	Chr8	22691471	36(A)	2314(T)	0.030169	2(T)	1290(A)	0.003091	0.9667 LOC_Os08g36000.1:(-),up-1936bp
COLDF	No	Chr8	22691596	8(G)	2298(A)	0.006914	5(A)	1267(G)	0.007831	0.9852 LOC_Os08g36000.1:(-),up-2061bp
COLDF	No	Chr8	22691640	6(A)	2290(G)	0.005213	4(G)	1268(A)	0.00627	0.9885 LOC_Os08g36000.1:(-),up-2105bp
COLDF	No	Chr8	22691878	6(T)	2274(C)	0.005249	3(C)	1275(T)	0.004684	0.9901 LOC_Os08g36000.1:(-),up-2343bp
COLDF	No	Chr8	22691902	6(C)	2284(T)	0.005226	2(T)	1274(C)	0.00313	0.9916 LOC_Os08g36000.1:(-),up-2367bp
COLDF	jiS	Chr8	22691950	4(C)	2362(T)	0.003376	0(N)	1304(C)	0	0.9966 LOC_Os08g36000.1:(-),up-2415bp
COLDF	No	Chr8	22691971	6(A)	2372(G)	0.005034	2(G)	1306(A)	0.003053	0.9919 LOC_Os08g36000.1:(-),up-2436bp
COLDF	No	Chr8	22691981	6(T)	2380(C)	0.005017	2(C)	1306(T)	0.003053	0.9919 LOC_Os08g36000.1:(-),up-2446bp
COLDF	No	Chr8	22691994	6(C)	2384(T)	0.005008	2(T)	1306(C)	0.003053	0.9919 LOC_Os08g36000.1:(-),up-2459bp
COLDF	No	Chr8	22691996	6(C)	2388(T)	0.005	2(T)	1308(C)	0.003049	0.9919 LOC_Os08g36000.1:(-),up-2461bp
COLDF	No	Chr8	22692094	6(A)	2392(G)	0.004992	4(G)	1306(A)	0.006088	0.9889 LOC_Os08g36000.1:(-),up-2559bp
qCTS-9	jS	Chr9	14528518	809(C)	1499(T)	0.455311	0(N)	1246(C)	0	0.4804 LOC_Os09g24440.1:(+),up-2672bp
qCTS-9	jS	Chr9	14528528	813(G)	1515(A)	0.454535	0(N)	1276(G)	0	0.4819 LOC_Os09g24440.1:(+),up-2662bp
qCTS-9	No	Chr9	14528573	111(G)	2257(A)	0.089355	2(A)	1278(G)	0.00312	0.9072 LOC_Os09g24440.1:(+),up-2617bp
qCTS-9	No	Chr9	14529420	2(A)	2250(G)	0.001775	377(G)	925(A)	0.411425	0.5488 LOC_Os09g24440.1:(+),up-1770bp
qCTS-9	No	Chr9	14529455	2(T)	2400(C)	0.001664	377(C)	927(T)	0.411051	0.5495 LOC_Os09g24440.1:(+),up-1735bp
qCTS-9	No	Chr9	14529489	115(A)	2293(G)	0.090953	2(G)	1306(A)	0.003053	0.9057 LOC_Os09g24440.1:(+),up-1701bp
qCTS-9	jS	Chr9	14529870	876(C)	1532(T)	0.462892	0(N)	1310(C)	0	0.466 LOC_Os09g24440.1:(+),up-1320bp
qCTS-9	No	Chr9	14529871	2(T)	2406(C)	0.00166	383(C)	925(T)	0.414147	0.545 LOC_Os09g24440.1:(+),up-1319bp
qCTS-9	jS	Chr9	14529878	876(G)	1534(A)	0.462728	0(N)	1310(G)	0	0.4664 LOC_Os09g24440.1:(+),up-1312bp
qCTS-9	No	Chr9	14530032	132(A)	2268(G)	0.10395	2(G)	1308(A)	0.003049	0.8926 LOC_Os09g24440.1:(+),up-1158bp
qCTS-9	No	Chr9	14530138	4(A)	2384(G)	0.003344	373(G)	931(A)	0.408445	0.5516 LOC_Os09g24440.1:(+),up-1052bp
qCTS-9	No	Chr9	14530140	2(C)	2386(G)	0.001674	375(G)	929(C)	0.409753	0.5513 LOC_Os09g24440.1:(+),up-1050bp
qCTS-9	No	Chr9	14530157	2(C)	2304(A)	0.001733	10(A)	936(C)	0.020918	0.9773 LOC_Os09g24440.1:(+),up-1033bp
qCTS-9	No	Chr9	14530214	112(T)	2284(C)	0.089119	2(C)	1306(T)	0.003053	0.9075 LOC_Os09g24440.1:(+),up-976bp
qCTS-9	No	Chr9	14530269	114(T)	2290(G)	0.090344	2(G)	1306(T)	0.003053	0.9063 LOC_Os09g24440.1:(+),up-921bp
qCTS-9	No	Chr9	14530395	140(A)	1982(G)	0.123245	2(G)	1306(A)	0.003053	0.873 LOC_Os09g24440.1:(+),up-795bp

qCTS-9	jS	Chr9	14530520	871(C)	1539(T)	0.461586	0(N)	1308(C)	0	0.4686	LOC_Os09g24440.1:(+),up-670bp
qCTS-9	No	Chr9	14530528	115(T)	2293(C)	0.090953	2(C)	1304(T)	0.003058	0.9057	LOC_Os09g24440.1:(+),up-662bp
qCTS-9	No	Chr9	14530547	2(A)	2408(G)	0.001658	384(G)	926(A)	0.414409	0.5447	LOC_Os09g24440.1:(+),up-643bp
qCTS-9	jS	Chr9	14530630	852(C)	1538(T)	0.458807	0(N)	1298(C)	0	0.4739	LOC_Os09g24440.1:(+),up-560bp
qCTS-9	jS	Chr9	14530857	863(C)	1521(A)	0.46191	0(N)	1296(C)	0	0.468	LOC_Os09g24440.1:(+),up-333bp
qCTS-9	No	Chr9	14530902	2(C)	2364(T)	0.001689	373(T)	915(C)	0.411461	0.5489	LOC_Os09g24440.1:(+),up-288bp
qCTS-9	No	Chr9	14530932	106(A)	2260(G)	0.085588	4(G)	1270(A)	0.00626	0.9079	LOC_Os09g24440.1:(+),up-258bp
qCTS-9	No	Chr9	14532090	115(G)	2295(A)	0.090882	3(A)	1307(G)	0.00457	0.9042	LOC_Os09g24440.1:(+),intron-1
qCTS-9	No	Chr9	14533332	115(C)	2295(A)	0.090882	2(A)	1308(C)	0.003049	0.9058	LOC_Os09g24440.1:(+),intron-1
qCTS-9	No	Chr9	14534121	115(T)	2295(A)	0.090882	2(A)	1308(T)	0.003049	0.9058	LOC_Os09g24440.1:(+),ACT->ACA,T->T,syn,exon-2
qCTS-9	No	Chr9	14535039	2(C)	2408(T)	0.001658	384(T)	926(C)	0.414409	0.5447	LOC_Os09g24440.1:(+),intron-4
qCTS-9	No	Chr9	14535961	114(A)	2292(T)	0.090273	2(T)	1308(A)	0.003049	0.9064	LOC_Os09g24440.1:(+),intron-6
qCTS-9	No	Chr9	14536300	2(A)	2408(G)	0.001658	385(G)	923(A)	0.41541	0.5432	LOC_Os09g24440.1:(+),AAT->AGT,N->S,no-syn,exon-7
qCTS-9	No	Chr9	14536351	113(A)	2295(T)	0.08945	2(T)	1304(A)	0.003058	0.9072	LOC_Os09g24440.1:(+),intron-7
qCTS-9	jS	Chr9	14536991	871(T)	1535(A)	0.461918	0(N)	1306(T)	0	0.468	LOC_Os09g24440.1:(+),intron-8
qCTS-9	jS	Chr9	14536993	871(G)	1539(A)	0.461586	0(N)	1306(G)	0	0.4686	LOC_Os09g24440.1:(+),intron-8
qCTS-9	No	Chr9	14537324	115(G)	2289(T)	0.091097	2(T)	1302(G)	0.003063	0.9055	LOC_Os09g24440.1:(+),TTG->TTT,L->F,no-syn,exon-9
qCTS-9	No	Chr9	14537525	115(G)	2285(A)	0.091241	2(A)	1308(G)	0.003049	0.9054	LOC_Os09g24440.1:(+),TGC->TAC,C->Y,no-syn,exon-10
qCTS-9	No	Chr9	14538416	115(T)	2291(C)	0.091025	2(C)	1308(T)	0.003049	0.9056	LOC_Os09g24440.1:(+),intron-12
qCTS-9	No	Chr9	14538451	2(T)	2402(A)	0.001663	382(A)	928(T)	0.413141	0.5465	LOC_Os09g24440.1:(+),intron-12
qCTS-9	jS	Chr9	14539347	871(T)	1539(C)	0.461586	0(N)	1310(T)	0	0.4686	LOC_Os09g24440.1:(+),intron-13
qCTS-9	No	Chr9	14540999	2(T)	2408(C)	0.001658	384(C)	926(T)	0.414409	0.5447	LOC_Os09g24440.1:(+),intron-15
qCTS-9	jS	Chr9	14541088	875(C)	1533(T)	0.462666	0(N)	1310(C)	0	0.4665	LOC_Os09g24440.1:(+),intron-15
qCTS-9	No	Chr9	14541528	4(T)	2404(G)	0.003317	381(G)	929(T)	0.412504	0.5458	LOC_Os09g24440.1:(+),intron-15
qCTS-9	jS	Chr9	14541695	874(G)	1536(A)	0.462273	0(N)	1308(G)	0	0.4673	LOC_Os09g24440.1:(+),intron-16
qCTS-9	jiS	Chr9	14542652	0(N)	2216(T)	0	0(N)	1310(A)	0	1	LOC_Os09g24440.1:(+),intron-17
qCTS-9	jS	Chr9	14543033	876(G)	1532(A)	0.462892	0(N)	1310(G)	0	0.466	LOC_Os09g24440.1:(+),intron-19
qCTS-9	No	Chr9	14543114	114(T)	2292(C)	0.090273	2(C)	1306(T)	0.003053	0.9064	LOC_Os09g24440.1:(+),intron-19
qCTS-9	No	Chr9	14543344	115(G)	2289(A)	0.091097	2(A)	1306(G)	0.003053	0.9055	LOC_Os09g24440.1:(+),intron-19
qCTS-9	No	Chr9	14543444	2(T)	2406(G)	0.00166	381(G)	927(T)	0.412876	0.5469	LOC_Os09g24440.1:(+),intron-19
qCTS-9	jS	Chr9	14544410	867(T)	1535(G)	0.46133	0(N)	1310(T)	0	0.4691	LOC_Os09g24440.1:(+),intron-21
qCTS-9	jS	Chr9	14544548	868(A)	1536(G)	0.461394	0(N)	1310(A)	0	0.469	LOC_Os09g24440.1:(+),intron-21
qCTS-9	No	Chr9	14544945	2(A)	2404(G)	0.001661	383(G)	927(A)	0.413777	0.5456	LOC_Os09g24440.1:(+),intron-23
qCTS-9	No	Chr9	14545663	24(G)	2384(C)	0.019735	385(C)	923(G)	0.41541	0.5261	LOC_Os09g24440.1:(+),intron-25
qCTS-9	jS	Chr9	14545684	870(T)	1538(G)	0.461522	0(N)	1310(T)	0	0.4687	LOC_Os09g24440.1:(+),intron-25
qCTS-9	No	Chr9	14546032	2(T)	2408(C)	0.001658	385(C)	925(T)	0.41504	0.5437	LOC_Os09g24440.1:(+),intron-25
qCTS-9	No	Chr9	14546503	873(G)	1537(A)	0.462045	1(A)	1309(G)	0.001526	0.4663	LOC_Os09g24440.1:(+),AGT->AAT,S->N,no-syn,exon-26
qCTS-9	No	Chr9	14547419	115(G)	2293(T)	0.090953	2(T)	1308(G)	0.003049	0.9057	LOC_Os09g24440.1:(+),TCG->TCT,S->S,syn,exon-26
qCTS-9	No	Chr9	14547460	115(T)	2295(C)	0.090882	2(C)	1308(T)	0.003049	0.9058	LOC_Os09g24440.1:(+),ATT->ACT,I->T,no-syn,exon-26

qCTS-9	jS	Chr9	14547510	876(A)	1534(G)	0.462728	0(N)	1310(A)	0	0.4664	LOC_Os09g24440.1:(+),ACA->GCA,T->A,no-syn,exon-26
