# Supplementary material for: High‐throughput selective sweep SNP‐guided cloning of cold‐tolerance genes in rice
Source: Plant Biotechnol J. 2024 Mar 7;22(8):2104–6. doi: 10.1111/pbi.14329 (PMC11258967; doi:10.1111/pbi.14329)
Supplement: Supplementary file 2 — Table S2 The genomic regions and selected candidate genes in the 11 mapped cold‐tolerance QTLs. [file PBI-22-2104-s005.pdf]

**Supporting Table S2.** The genomic regions and selected candidate genes in the 11 mapped cold-tolerance QTLs

| QTL | Developmental Stage | Reference | Chromosome | Location | Number of the genes predicted | Candidate |
|---|---|---|---|---|---|---|
| *qLTSSvR2-2* | Germination | Pan et al., 2015 | 2 | 4,313,914-6,315,036 | 298 | *CT-1* |
| *qLTG3-1* | Germination | Fujino et al., 2008 | 3 | 1-1,220,919 | 195 | *CT-2* |
| *qCT-3-2* | Booting | Zhu et al., 2015 | 3 | 824,682-3,150,858 | 373 | *CT-3*, *4*, *5*, *6*, *7*, *8* |
| *qCTSS-5* | Seedling | Yang et al., 2013 | 5 | 20,280,000-29,960,000 | 1605 | CT-*9*, *10*, *11* |
| *L92/qCTS9-5* | Seedling | Lv et al., 2016; Wang et al., 2016 | 9 | 3,304,471- 5,488,259 | 303 | *CT-12* |
| *qPLR-9-4* | Germination | Thapa et al., 2020 | 9 | 15,325,535-17,325,535 | 283 | *CT-13* |
| *qCTB10-2* | Booting | Xu et al., 2008 | 10 | 5,438,607-7,571,107 | 297 | *CT-14*, *15*, *16*, *17*, *18*, *19* |
| *qSCT11* | Seedling | Kim et al., 2014 | 11 | 21,301,763-23,303,659 | 279 | *CT-20* |
| *L22* | Seedling | Lv et al., 2016 | 2 | 13,548,326-15,729,038 | 305 | *CT-21* |
| *qLTRSSR3-1* | Booting | Pan et al., 2015 | 3 | 2,678,950-4,680,068 | 343 | *CT-22*, *23*, *24*, *25* |
| *qCTSSR1-1* | Booting | Pan et al., 2015 | 1 | 7,075,257-9,075,304 | 307 | *CT-26*, *27* |
