# Supplementary material for: High‐throughput selective sweep SNP‐guided cloning of cold‐tolerance genes in rice
Source: Plant Biotechnol J. 2024 Mar 7;22(8):2104–6. doi: 10.1111/pbi.14329 (PMC11258967; doi:10.1111/pbi.14329)
Supplement: Supplementary file 3 — Table S3 The candidate genes and their H p and F ST information in the 11 cold‐tolerance QTLs. [file PBI-22-2104-s001.pdf]

Supporting Table S3. The candidate genes and their H_p and F_{ST} information in the 11 cold-tolerance QTLs

**QTL**	**Candidate**	**MSU gene ID**	**Gene description**	**SNP position**	**Allele-*japonica***	**Allele-*indica***	**Amino acid variation**	F_{ST}	H_p**-*japonica***	H_p**-*indica***	**Selection type**	Haplotype-japonica	H_p-japonica (haplotype)	Haplotype-indica	H_p-indica (haplotype)
qLTSSvR2-2	*CT-1*	LOC_Os02g09510	Limonoid UDP-glucosyltransferase	4889711	A	G	L150P	0.807900	0.000000	0.189700	*j* S	TTCAGCACTCACGCGGCC<br>TGCGAGACTA	0.000000	CCTGATGGATGTATCATTC<br>ATAGCCTCT<br>CCTGATGGATACGCGGCC<br>TGCGAGACTA<br>CCTGACGGATGTATCATTC<br>ATAGCCTCT	0.486297
qLTG3-1	*CT-2*	LOC_Os03g01910	Transcription factor BTF3	560608<br>560609<br>560735	C<br>A<br>G	T<br>G<br>A	V169A<br>V112A<br>A127V	0.941900<br>0.943500<br>0.941900	0.000000<br>0.000000<br>0.000000	0.057966<br>0.056404<br>0.057966	*j* S	CGCTGCAGGCCTCTACGG<br>TATGCCCTAAAAGCCTCT<br>GAAGGATCCAGC<br>TGCTGCAGGCCTCTACGG<br>TATGCCCTAAAAGCCTCT<br>GAAGGTTCCAGC<br>CGCTGCAGGCCTCTACGG<br>TCTGCCCTAAAAGCCTCT<br>GAAGGATCCAGC	0.097519	TAACCTGAATAAAGGTTA<br>ACCAGTTCGGGCAATAAC<br>ACGAATCTTGAT<br>TGACGTGAATCAAGGTTA<br>ACCAGTTCGGGCAATAAC<br>ACAAATCCTGAC<br>TGACCTGAATCAAGGTTA<br>ACCAGTTCGGGCAATAAC<br>ACAAATCCTGAC	0.172667
qCT-3-2	*CT-3*	LOC_Os03g03034	Flavonol synthase/flavanone 3-hydroxylase	1244354	G	T	P145T	0.811000	0.000000	0.186742	*j* S	CGTCGCGGGTAGAAGATA<br>CGACAACTGTG<br>CGTCGCGGGTAGGGGACG<br>CGGCGGCTGCG<br>CGTCGCGGGTAGGGGACG<br>CAGCGGCTGCG	0.498706	TTCTAGTCACCCGGAGCGT<br>AGTGGTCATT<br>TTCTACGCATACGGAACGT<br>AGCGGTCGTT<br>TTCTAGTCATACGGAACGT<br>AGCGGTCGTT	0.206048
qCT-3-2	*CT-4*	LOC_Os03g05320		2582653	A	G	K197R	0.827600	0.000000	0.170638	*j* S	CTGTAG<br>CTGTAA<br>ATGTAG	0.003072	AGACGA<br>CTGTAG<br>AGGCGA	0.171882
qCT-3-2	*CT-5*	LOC_Os03g05330	HEAT repeat family protein	2598158<br>2594469<br>2599113<br>2605230	C<br>A<br>A<br>C	A<br>G<br>G<br>A	P308T<br>K97R<br>H439R<br>P1059Q	0.824900<br>0.827000<br>0.826700<br>0.826300	0.000000<br>0.000000<br>0.000000<br>0.000000	0.173327<br>0.171311<br>0.171566<br>0.171984	*j* S	TAAACACCAAGCGCCA<br>TAGACACCAAGCGCCA<br>TAAACACCAGGCGCCA	0.003072	CGGGAGTTGGAGAATG<br>TAAACACCAAGCGCCA<br>CGAGCGTTGGGGAATG	0.172401

qCT-3-2	*CT-6*	LOC_Os03g0<br>5370	expressed protein	2639298<br>2639433<br>2639815<br>2639955<br>2640039<br>2640117<br>2640126	C<br>C<br>G<br>A<br>G<br>T<br>C	T<br>T<br>A<br>G<br>A<br>A<br>T	R10 stop_gained<br>Q55 stop_gained<br>R130H<br>S177G<br>V205M<br>L231M<br>Q234 stop_gained	0.878300<br>0.797600<br>0.878200<br>0.878300<br>0.797600<br>0.878300<br>0.796300	0.000000<br>0.000000<br>0.000000<br>0.000000<br>0.000000<br>0.000000<br>0.000000	0.121081<br>0.199607<br>0.121174<br>0.121081<br>0.199607<br>0.121081<br>0.200892	*j* S	CTCCGAGTC	0.000000	TCTTAGAAT<br>CTCCGAGTC<br>TTCTAGGAC	0.200832
qLTRSSR3-1	*CT-7*	LOC_Os03g0<br>5520	Nicotiana lesion-<br>inducing like	2740113	C	T	D109N	0.780800	0.000000	0.215627	*j* S	GCCCCGGGAAGCCATGCA<br>TCACTCGATAGGACGTGG<br>ACAGTGCCAAGCATAG<br>GCCCCGGGAAGCCATGCA<br>TCGCTCGATAGGACGTGG<br>ACAGTGCCAAGCATAG<br>ACCCCGGAGGGCCGTGCA<br>CCGTACGATGGGGCGCGG<br>ACAGCACCAAGCGTAG	0.190446	ATTTTAAAGGAGTGCAGC<br>CTGTATATCGAAGTACATG<br>TTACATTGGATGCGA<br>GCCCCGGGAAGCCATGCA<br>TCACTCGATAGGACGTGG<br>ACAGTGCCAAGCATAG<br>ATTTTAAAGGAGTGCAGC<br>CTGTATATCGAAGTACATG<br>TTACATTGGATACGA	0.244898
qLTRSSR3-1	*CT-8*	LOC_Os03g0<br>5450	expressed protein	2695998<br>2696314<br>2696460<br>2696462	G<br>T<br>A<br>T	C<br>G<br>G<br>C	T138R<br>Q63P<br>T14A<br>T14A	0.977300<br>0.978600<br>0.978800<br>0.978800	0.022639<br>0.021404<br>0.021178<br>0.021210	0.000000<br>0.000000<br>0.000000<br>0.000000	*ji* S	GTGATAGTGGATGGTCTG<br>CCACCTAA<br>CGGGCAGCAAATGGGCTA<br>CCACCAGC<br>GTGATAGTGGATGGTCTG<br>CCACTTAA	0.023563	CGTGCTTCAATCTAGAGAT<br>TGTTAGC<br>CGTGCATCAATCGAGAGA<br>TTGCCAGC<br>CGTGCATCAAACGAGAGA<br>CCGCCAGC	0.101763
qCTSS-5	*CT-9*	LOC_Os05g4<br>4900	expressed protein	26095560	G	C	V166L	0.949600	0.000000	0.050291	*j* S	CTAGATAGCTCGT<br>CTAGATAGTTCGT<br>CTAGATAGCCCGT	0.064638	AGGATAGCTCGCC<br>AGGATTGCTCGGC<br>CTAATTGGTCGGT	0.041683
qCTSS-5	*CT-10*	LOC_Os05g4<br>3130	Brix domain-<br>containing protein<br>1	25043157<br>25043187	C<br>A	T<br>C	R59K<br>L49W	0.995000<br>0.995000	0.000000<br>0.000000	0.004967<br>0.004971	*ji* S	A<br>T	0.003049	T<br>A	0.005802

qCTSS-5	*CT-11*	LOC_Os05g43140	expressed protein	25044944<br>25044750	A<br>A	G<br>G	T72A<br>H7R	0.996600<br>0.995000	0.000000<br>0.000000	0.003356<br>0.005025	*ji* S	AAGGAATCT<br>TAGGAATCT	0.001584	TGCAGGCTG	0.000000
qCTB10-2	*CT-12*	LOC_Os10g10480	expressed protein	5762406	G	A	G611S	0.394600	0.000000	0.491295	*j* S	GAAAG<br>AGTAG<br>GGTAG	0.376973	AGTGA<br>GAAGA<br>AGTAA	0.483783
qSCT11	*CT-13*	LOC_Os11g37290	expressed protein	22019409	C	T	A117T	0.473500	0.000000	0.459032	*j* S	ACG<br>GCG	0.339338	GTA<br>GCG<br>GCA	0.442889
L22	*CT-14*	LOC_Os02g25650	expressed protein	15004117	C	T	M1I	0.993900	0.006088	0.000000	*ji* S	CCATTGCAGCCGATGGAG<br>GGATGTAATGATTG<br>CCATCGCAGCCTATGGAG<br>GGATGTAATGATTG<br>CCATTGCAGCCGCTGGAG<br>GGATGTAATGATTG	0.499922	TTGCCATGATTTCCAAGAC<br>AGCACGCCAGGCA<br>TTGCCATGACTTCCAAGAC<br>AGCACGCCAGGCA	0.016934
L22	*CT-15*	LOC_Os02g25780	expressed protein	15106620	A	G	I94V	0.989700	0.010327	0.000000	*ji* S	TCGGTATCAGAGATGTAA<br>TG<br>TTGGTAGTGGGGATGCAG<br>TG<br>TCGGTATCAGAGATGCAA<br>TG	0.015720	GTAACGGTGAGAGCACCG<br>CA<br>GTGACGGCGAGAGCACAG<br>CA<br>TTAACGGTGAGAGCACCG<br>CA	0.023703
L22	*CT-16*	LOC_Os02g25830	expressed protein	15134482	A	C	E161A	0.984800	0.015151	0.000000	*ji* S	ATGAGCGGGCACCTTGAA<br>ATGGGGGGGCACCTTGAC<br>ATGGGCGGGCACCTTGAA	0.007746	GCCGAGCAATGTTAATGC<br>GTGGAGCAACGCTAATGC<br>GTCGAGCAACGCTAATGC	0.024934
L22	*CT-17*	LOC_Os02g25840	Expression of conserved oligomeric Golgi complex component 4	15142849	T	C	N212D	0.993900	0.006135	0.000000	*ji* S	GCC<br>TTT<br>TCC	0.007616	TTT<br>TCT<br>GCT	0.087674

L22	*CT-18*	LOC_Os02g25850	Transposon protein, Pong sub-class	15145039<br>15145285<br>15145833	G<br>A<br>G	A<br>G<br>T	E4K<br>T86A<br>E243 stop_gained	0.993900<br>0.879900<br>0.992400	0.006088<br>0.118689<br>0.007604	0.000000<br>0.000830<br>0.000000	*ji* S	GAG<br>GGG<br>AGT	0.120021	AGT<br>AAT	0.000830
L22	*CT-19*	LOC_Os02g25890	expressed protein	15181056<br>15181183	T<br>A	C<br>G	F73L<br>K115R	0.984800<br>0.993900	0.015174<br>0.006088	0.000000<br>0.000000	*ji* S	TCCATAC<br>TCCACAC<br>CATGCGT	0.018180	CATGCGT<br>CCTACGT<br>CATACGT	0.024647
L92/qCTS9-5	*CT-20*	LOC_Os09g08150	expressed protein	4226063<br>4226080<br>4226134	C<br>C<br>G	A<br>T<br>A	V77L<br>R71H<br>A53V	0.343600<br>0.343600<br>0.344700	0.000000<br>0.000000<br>0.000000	0.499710<br>0.499710<br>0.499646	*j* S	CCGTCGGTTTAGACATGACGCCTTATTA<br>CCGTCGGTTTAGACAAGATCCCTTAGCA<br>CCGTCGGTTTAGACATGATGCCTTAGTA	0.419876	CCGTCGGTTCAAGTGAGATCCCTTAGCA<br>ATACGAACCCGAGTGAAGTCGTCCGGCG<br>CCGTCGGTCCAAGTGAGATCGTTTAGCA	0.499902
qLTRSSR3-1	*CT-21*	LOC_Os03g06470	expressed protein	3238650	C	G	D59H	0.996700	0.000000	0.003317	*ji* S	CTA	0.000000	GCT<br>CTT<br>GTT	0.004979
qPLR-9-4	*CT-22*	LOC_Os09g26650	2Fe-2S iron-sulfur cluster binding domain containing protein	16171299	C	T	A12V	0.806500	0.000000	0.191093	*j* S	TGATGTATAGATTTGTATGGGGAGACCCTTCC<br>TGGTGTATAGATTTGTATGGGGAGACCCTTCC<br>TGGCGTATAGATTTGTATGGGGAGACCCTTCC	0.499975	CAGCAGTCGCGCCAACCCCAAAGAGGATGCTT<br>CAGCGGTCGCGCCAATACCAGAGGACACGCCT<br>CGGCGGATGGGCCTGTACCGGAGGGCACGCCC	0.169768
qPLR-9-4	*CT-23*	LOC_Os09g26700	Choline/ethanolamine kinase	16225212	G	A	G15E	0.843500	0.000000	0.155153	*j* S	GTGACGGAAGGGGTGATAACGGTCTATGACCCC<br>GTGACGGAAGGGGTGATAACGGTCTGTGACCCC	0.004566	AATCTAATTTAAACATCTCAAAGGCGCACTTGT<br>AATCTAATTTAAATATCTCAAAGGCGCACTTGT<br>AATCTAATTTAAACATCTAAGGGGCGCGCCTCT	0.010989
qPLR-9-4	*CT-24*	LOC_Os09g26730	Chaperonin	16239650	C	T	V83I	0.959300	0.000000	0.040633	*ji* S	AGCCGCA<br>ATCCGCA<br>AGTCGCA	0.006116	GTTTAAG<br>GTTTACA<br>GTTTAAA	0.141229

qPLR-9-4	*CT-25*	LOC_Os09g26780	Zinc-finger protein	16274847	T	G	E89D	0.961100	0.000000	0.038803	*ji* S	AGTTTACATTAAATCATG CTGTACACCATTA<br>AGTTCGCGTGTGTCCGCG TTAAACACCGCCG	0.003205	GTCGCGTGCGTGTCTGCAT AAAGTGTTGCCG<br>ATTGCGCGCGTGTCTGCAT TAAGCGCTGCCG<br>AGTTCGCGTGTGTCCGCGT TAAACACCGCCG	0.149628
qCTSSR1-1	*CT-26*	LOC_Os01g13229	Cyclin-A1	7369756	T	G	L654F	0.923600	0.076238	0.000000	*ji* S	TGAGTTCCCAACATGAAA AAAT<br>TGAGTTCCCAACATAAAA AAAT<br>TGAGTTCCCAGCATGAAA AAAT	0.072708	GAGTGATATGGGGCACGT GGGC<br>GAATGATATGGGGCACGT GGGC<br>GAGTGATCTGGGGCACGT GGGC	0.100674
qCTSSR1-1	*CT-27*	LOC_Os01g13300	B3 DNA binding domain containing protein	7421797	A	C	I422L	0.919300	0.080444	0.000000	*ji* S	CTCCTCTTTTCATCGTCGC CCCTGCTCTCATACTTTTC CCGGAAATGT<br>CTCCTCTTTTCGTCGTCGC CCCTGCTCTCATACTTTTC CCGGAAATGT<br>TTCCTCTTTTCATCGTCGC CCCTGCTCTCATACTTTTC CCGGAAATGT	0.351643	TCTTCTCCCCTGCTACTTT TTTCATAACTGCGTCGCCA TTCAGCTGAC<br>TTCCCCCCCTCGCTGCTGC CCCCGCTCCCGCGTCGCCC CCGAGCTGAC<br>TCTTCTCCCCTGCTACTTT TTTCATAACTGCATCGCCA TTCAGCTGAC	0.104184

H_P, pooled heterozygosity; F_{st}, fixation statistic
