# Supplementary material for: High‐throughput selective sweep SNP‐guided cloning of cold‐tolerance genes in rice
Source: Plant Biotechnol J. 2024 Mar 7;22(8):2104–6. doi: 10.1111/pbi.14329 (PMC11258967; doi:10.1111/pbi.14329)
Supplement: Supplementary file 4 — Table S4 The haplotypes in the coding regions of the 27 SSNP‐selected candidate genes. [file PBI-22-2104-s004.pdf]

Supporting Table S4. The haplotypes in the coding regions of the 27 SSNP-selected candidate genes

Candidate	Haplotypes	SNPs	Sample List	Group
	Hap1	GTTACTGATG	295	XI: 288<br>admix: 7
	Hap2	ATCGTCAGCG	276	Aus: 6<br>Bas: 3<br>GJ: 237<br>XI: 26<br>admix: 4
	Hap3	GTCGTCAGCG	210	GJ: 141<br>XI: 65<br>admix: 4
	Hap4	-TTACTGATG	187	XI: 182<br>admix: 5
	Hap5	GTTACCGACA	168	Aus: 43<br>Bas: 14<br>GJ: 1<br>XI: 100<br>admix: 10
	Hap6	-TCGTCAGCG	143	GJ: 106<br>XI: 35<br>admix: 2
	Hap7	-TTACCGACA	106	Aus: 27<br>Bas: 13<br>XI: 64<br>admix: 2
	Hap8	GTTACTGACG	81	XI: 81
	Hap9	-TTACTGACG	62	Aus: 1<br>XI: 60<br>admix: 1
	Hap10	----------	25	Aus: 4<br>Bas: 3<br>GJ: 4<br>XI: 12<br>admix: 2
CT-1	Hap11	--CGTCAGCG	24	GJ: 18<br>XI: 6
	Hap12	-TTACTGA-G	23	XI: 21<br>admix: 1<br>na: 1
	Hap13	GTCGCCGGCG	23	Aus: 5<br>XI: 17<br>admix: 1
	Hap14	AKCGTCAGCG	19	Aus: 1<br>GJ: 15<br>XI: 3
	Hap15	GKTACTGATG	19	XI: 19
	Hap16	--TACCGACA	19	Aus: 9<br>Bas: 1<br>XI: 8<br>admix: 1
	Hap17	-TTACCGAC-	19	Aus: 4<br>Bas: 2<br>XI: 12<br>admix: 1
				Aus: 2

	Hap18	---A--GA--	17	GJ: 1<br>XI: 13
	Hap19	-TTACTGA--	17	XI: 17
	Hap20	--TACTGATG	17	XI: 17
	Hap21	-TCGCCGGCG	16	Aus: 7<br>XI: 8<br>admix: 1
	Hap22	---A-TGA--	15	GJ: 1<br>XI: 13<br>admix: 1
CT-2	Hap1	TGAAAA	1824	Aus: 129<br>Bas: 4<br>GJ: 4<br>XI: 1651<br>admix: 35<br>na: 1
	Hap2	CAGGGT	1072	Aus: 66<br>Bas: 64<br>GJ: 825<br>XI: 78<br>admix: 39
	Hap3	YRRRRW	34	Bas: 1<br>GJ: 2<br>XI: 13<br>admix: 18
CT-3	Hap1	CTGC	1359	Aus: 23<br>Bas: 20<br>XI: 1292<br>admix: 24
	Hap2	TGGC	844	Aus: 132<br>Bas: 31<br>GJ: 617<br>XI: 33<br>admix: 31
	Hap3	TGGS	237	Aus: 12<br>Bas: 9<br>GJ: 197<br>XI: 11<br>admix: 8
	Hap4	CTGS	186	Aus: 5<br>Bas: 4<br>XI: 172<br>admix: 5
	Hap5	CGAC	166	Aus: 4<br>XI: 159<br>admix: 2<br>na: 1
	Hap6	CGGC	32	Aus: 12<br>Bas: 2<br>GJ: 3<br>XI: 7

				admix: 8
CT-4	Hap1	CCG	1802	Aus: 192<br>Bas: 43<br>GJ: 3<br>XI: 1519<br>admix: 44<br>na: 1
	Hap2	CTA	1041	Aus: 2<br>Bas: 23<br>GJ: 826<br>XI: 159<br>admix: 31
	Hap3	CYR	38	GJ: 8<br>XI: 15<br>admix: 15
	Hap4	ACG	36	XI: 32<br>admix: 4
	Hap5	-CG	28	Aus: 2<br>Bas: 2<br>XI: 24
CT-5	Hap1	GAGCTTGGAGACA	1609	Aus: 54<br>Bas: 37<br>GJ: 2<br>XI: 1484<br>admix: 31<br>na: 1
	Hap2	ACACCCAAGCGCC	744	Aus: 3<br>Bas: 18<br>GJ: 580<br>XI: 123<br>admix: 20
	Hap3	ACACCCAAGCGTC	240	Bas: 2<br>GJ: 203<br>XI: 26<br>admix: 9
	Hap4	GAGATTGGAGACA	162	Aus: 130<br>Bas: 4<br>XI: 18<br>admix: 10
	Hap5	RMRCYYRRRSRCM	17	GJ: 3<br>XI: 5<br>admix: 9
	Hap1	TCATCGTAGAATC	1613	Aus: 127<br>Bas: 35<br>GJ: 1<br>XI: 1412<br>admix: 37<br>na: 1
	Hap2	CTACCGCGAGTCC	885	Aus: 2<br>Bas: 25<br>GJ: 713<br>XI: 115<br>admix: 30
				Aus: 1<br>Bas: 2

	Hap3	CTMCCGCGAGTCC	117	GJ: 103<br>XI: 9<br>admix: 2
CT-6	Hap4	TCMTCGTAGAATC	90	Aus: 7<br>XI: 81<br>admix: 2
	Hap5	TTACGTTAGGACT	59	GJ: 1<br>XI: 58
	Hap6	TTATCGTAGAATC	54	Aus: 43<br>Bas: 6<br>XI: 3<br>admix: 2
	Hap7	YYAYCGYRRRWYC	21	GJ: 2<br>XI: 7<br>admix: 12
	Hap8	T-ATCGTAGAATC	20	Aus: 2<br>Bas: 1<br>XI: 16<br>admix: 1
	Hap9	TYATCGTAGAATC	15	Aus: 2<br>XI: 12<br>admix: 1
	Hap1	TTA	1424	Aus: 27<br>Bas: 3<br>GJ: 1<br>XI: 1373<br>admix: 19<br>na: 1
	Hap2	CCG	1173	Aus: 25<br>Bas: 32<br>GJ: 828<br>XI: 244<br>admix: 44
CT-7	Hap3	TCA	291	Aus: 142<br>Bas: 36<br>XI: 102<br>admix: 11
	Hap4	YYR	34	GJ: 3<br>XI: 20<br>admix: 11
	Hap5	TYA	18	Aus: 1<br>Bas: 1<br>XI: 10<br>admix: 6
	Hap1	CGGGTGC	1663	Aus: 3<br>Bas: 8<br>GJ: 1<br>XI: 1626<br>admix: 24<br>na: 1
	Hap2	GGGTGAT	870	Aus: 3<br>Bas: 13<br>GJ: 796<br>XI: 29<br>admix: 29
	Hap3	CGAGTGC	175	Aus: 131<br>Bas: 7<br>XI: 26

CT-8				admix: 11
	Hap4	CAGGTGC	82	Aus: 45<br>Bas: 29<br>XI: 6<br>admix: 2
	Hap5	CGGGGGC	24	Aus: 4<br>Bas: 9<br>GJ: 5<br>XI: 1<br>admix: 5
	Hap6	SGGKKRY	20	GJ: 4<br>XI: 6<br>admix: 10
	Hap7	CGGGT--	15	Aus: 1<br>XI: 13<br>admix: 1
CT-9	Hap1	C	1666	Aus: 6<br>Bas: 1<br>GJ: 5<br>XI: 1622<br>admix: 31<br>na: 1
	Hap2	G	1279	Aus: 192<br>Bas: 71<br>GJ: 834<br>XI: 130<br>admix: 52
	Hap3	S	64	Aus: 3<br>Bas: 2<br>GJ: 13<br>XI: 28<br>admix: 18
	Hap4	-	15	Bas: 2<br>GJ: 2<br>XI: 9<br>admix: 2
CT-10	Hap1	GTCATTCGTCCAA	1749	Aus: 175<br>Bas: 7<br>GJ: 8<br>XI: 1513<br>admix: 45<br>na: 1
	Hap2	AATGCATACATGG	974	Aus: 1<br>Bas: 64<br>GJ: 818<br>XI: 62<br>admix: 29
	Hap3	GTCATTCG-CCAA	34	Aus: 5<br>GJ: 1<br>XI: 28
	Hap4	GTCATTCGTCCA-	24	Aus: 2<br>XI: 21<br>admix: 1
	Hap5	RWYRYWYRYMYRR	18	GJ: 4<br>XI: 6<br>admix: 8
	Hap6	GWYATTCGTCCAA	16	Aus: 4<br>XI: 12

CT-11	Hap1	TAGGGCATCT	833	Aus: 1<br>Bas: 49<br>GJ: 710<br>XI: 49<br>admix: 24
	Hap2	TGGCACACTG	457	GJ: 1<br>XI: 443<br>admix: 13
	Hap3	TGGCRMACTG	209	GJ: 1<br>XI: 207<br>admix: 1
	Hap4	KGGCACACTG	173	GJ: 1<br>XI: 167<br>admix: 5
	Hap5	KAGGGCATCT	93	Bas: 7<br>GJ: 76<br>XI: 9<br>admix: 1
	Hap6	TGGCACRCTG	91	GJ: 1<br>XI: 90
CT-12	Hap1	GGTATGTCGGGCGCCTGGGGT<br>TTAATTCTGCGGGGGATCAGG<br>ACACCGATGGGCCAACTCCAT<br>TGATACTTGACC	479	Bas: 9<br>GJ: 410<br>XI: 48<br>admix: 12
	Hap2	GGTATGTCGGGCGCTTGGGGT<br>TTGGTTCTGCGGGGGACCAGG<br>GCACTGATGGGCAAATTCCAC<br>TGGTACTCATCC	443	Aus: 1<br>Bas: 1<br>XI: 431<br>admix: 10
	Hap3	GTTATGTCGGGACCCCGCAAG<br>GCGGTTTTACGAGGGTCCCCG<br>GCTCCATCAAGCAAACCTTTC<br>CGGCCTCCGAGC	421	Bas: 1<br>GJ: 2<br>XI: 415<br>admix: 3
	Hap4	GGTATGTCGGGCGCCTAGGGT<br>GTGGTTCTGCGGGGGACCAGG<br>GCACTGATGGGCAAACTCCAC<br>TGGTACTTGACC	224	Aus: 179<br>Bas: 1<br>GJ: 2<br>XI: 31<br>admix: 11
	Hap5	GGTTTGTCGGGCGCCTGCAAG<br>GCGGTGTTACGAGGGACCCCG<br>GCTCCATCAGTCAGACCTTTC<br>CGGCCTCCGACC	214	GJ: 86<br>XI: 126<br>admix: 2
	Hap6	GTTACGTCGGGACCCCGCAAG<br>GCGGTTTTACGAGGGTCCCCG<br>GCTCCATCAAGCAAACCTTTC<br>CGGCCTCCGAGC	150	Aus: 1<br>GJ: 1<br>XI: 145<br>admix: 3
	Hap7	GGTATGTCGGGCGCCTGGGGT<br>TTAATTCCGCGGGGGATCAGG<br>ACACCGATGGGCCAACTCCAT<br>TGATACTTGACC	110	GJ: 102<br>XI: 3<br>admix: 5
	Hap8	GGTTTGTCGGGCGCCTGCAAG<br>GCGGTGTAACGAGGGACCCC<br>GGCTCCATCAGTCAGACCTTT<br>CCGGCCTCCGACC	42	GJ: 1<br>XI: 41
	Hap9	GGTTTGTCGGGCGCCTGCAAG<br>GCGGTGTTACGAGGGACYCCG<br>GCTCCATCAGTCAGACCTTTC<br>CGGCCTCCGACC	41	GJ: 14<br>XI: 27
	Hap10	GTTATGTCGGTACCCCGCAAG<br>GCGGTTTTACGAGGGTCCCCG<br>GCTCCATCAAGCAAACCTTTC<br>CGGCCTCCGAGC	38	XI: 38
	Hap11	GCATATCAGGCCTCCGCAAGG<br>CGGCTTTACAAGGGACCCCGG<br>[illegible]	35	Bas: 22<br>XI: 7

	CTCCATCGGGCAAACCTTTCC<br>AGCCTCCGACC		admix: 6
Hap12	CGTATGATAGGCCCCCGCAAG<br>GCGGTTTTATGATGGACCCCG<br>GCTTCATCGGGCAATCCTTTC<br>CGGCCTCCGACC	28	GJ: 23<br>XI: 3<br>admix: 2
Hap13	GGTATGTCGGGCGCTTGGGGT<br>TTGGTTCTGCGGGGGACCAGG<br>GTACTGATGGGCAAATTCCAC<br>TGGTACTCATCC	25	XI: 25
Hap14	GGTATGTCGAGCGCCTGGGGT<br>TTGGTTCTGCGGGAAACCAGG<br>ACACCAATGGGTAAACTCCAC<br>TGGTACTTGACT	24	Aus: 1<br>Bas: 15<br>GJ: 3<br>XI: 1<br>admix: 4
Hap15	GGTTTGTCGGGCGCCTGCAAG<br>GGGTGTTACGAGGGACCCCGG<br>CTCCATCAGTCAGACCTTTCC<br>GGCCTCCGACC	22	GJ: 9<br>XI: 13
Hap16	GGTATGTCAGGCGCCTGGGGT<br>TTGGTTCTGCGGGGGACCAGG<br>ACACCGATGGGCAAACTCCAC<br>TGGTACTTGACC	22	GJ: 20<br>XI: 1<br>na: 1
Hap17	GGTTTGTCGGGCGCCTGCAAG<br>GCGTGTTACGAGGGACCCCGG<br>CTCCATCAGTCAGACCTTTCC<br>GGCCTCCGACC	21	GJ: 8<br>XI: 13
Hap18	CGTATGATAGGCCCCCGCAAG<br>GCGGTTTTACAAGGGACCCCA<br>GCTCCATCGGGCAAACCTTTC<br>CGGCCTCCGACC	16	GJ: 16
Hap19	CGTATGTCAGGCCCCCGCAAG<br>GCGGTTTTACAAGGGACCCCA<br>GCTCCATCGGGCAAACCTTTC<br>CGGCCTCCGACC	15	GJ: 14<br>admix: 1

Hap1	GGAGAGAC-----	431	Aus: 24<br>Bas: 2<br>GJ: 160<br>XI: 230<br>admix: 15
Hap2	GAAGAGAC-----	308	Bas: 1<br>GJ: 4<br>XI: 297<br>admix: 5<br>na: 1
Hap3	-------------	187	Aus: 48<br>Bas: 16<br>GJ: 30<br>XI: 81<br>admix: 12
Hap4	AGAGAGACCAGGG	123	GJ: 120<br>XI: 3
Hap5	AGAGAGAC-----	77	GJ: 74<br>XI: 1<br>admix: 2
Hap6	GGAGATAC-----	63	XI: 62<br>admix: 1
Hap7	GGAGAGACCAGGG	57	Aus: 2<br>GJ: 32<br>XI: 21<br>admix: 2
Hap8	GGAGCGAC-----	57	Aus: 35<br>GJ: 3<br>XI: 16<br>admix: 3
			Aus: 3

	Hap9	G---AGAC-----	56	Bas: 1<br>GJ: 10<br>XI: 41<br>admix: 1
	Hap10	GAAG---------	49	Bas: 3<br>XI: 43<br>admix: 3
	Hap11	G-AGAGAC-----	48	Aus: 2<br>Bas: 1<br>GJ: 14<br>XI: 29<br>admix: 2
	Hap12	GA--AGAC-----	46	Bas: 1<br>XI: 45
	Hap13	-AAGAGAC-----	42	XI: 41<br>admix: 1
CT-13	Hap14	GA-----------	40	XI: 40
	Hap15	GAAGA--------	38	Aus: 2<br>Bas: 2<br>XI: 34
	Hap16	G------------	37	Aus: 1<br>Bas: 2<br>GJ: 5<br>XI: 28<br>admix: 1
	Hap17	GAAG-GAC-----	27	XI: 27
	Hap18	GGAGAGACC----	24	Aus: 2<br>GJ: 12<br>XI: 10
	Hap19	GAAGAGACTTGGG	23	XI: 23
	Hap20	GGAGAGACTTGGG	23	Aus: 1<br>GJ: 2<br>XI: 19<br>admix: 1
	Hap21	G----GAC-----	21	Aus: 1<br>GJ: 6<br>XI: 13<br>admix: 1
	Hap22	AGAGAGATCAGGG	20	GJ: 20
	Hap23	GGAGAGACCAGGA	20	GJ: 14<br>XI: 6
	Hap24	-AAG---------	19	Bas: 1<br>XI: 18
	Hap25	-A--AGAC-----	19	XI: 18<br>admix: 1
	Hap26	-GAGAGAC-----	19	GJ: 17<br>XI: 2
	Hap27	----AGAC-----	19	Aus: 1<br>GJ: 1<br>XI: 16<br>admix: 1
	Hap28	GRAGAGAC-----	18	GJ: 1<br>XI: 15<br>admix: 2

	Hap29	GAAGAGACC----	17	XI: 17
	Hap30	GAAGAGACCAAGG	17	XI: 17
	Hap31	GGAGAGACCA---	16	Aus: 1<br>GJ: 6<br>XI: 9
CT-14	Hap1	TT	1893	Aus: 149<br>Bas: 6<br>GJ: 15<br>XI: 1686<br>admix: 36<br>na: 1
	Hap2	CC	933	Aus: 1<br>Bas: 67<br>GJ: 809<br>XI: 19<br>admix: 37
	Hap3	C-	83	Aus: 48<br>GJ: 1<br>XI: 32<br>admix: 2
	Hap4	YY	38	Bas: 1<br>GJ: 6<br>XI: 11<br>admix: 20
	Hap5	-T	17	Aus: 1<br>XI: 16
	Hap6	YC	15	Bas: 1<br>GJ: 12<br>admix: 2
	Hap1	CGCGTCA	893	Aus: 69<br>Bas: 1<br>GJ: 8<br>XI: 801<br>admix: 14
	Hap2	AGCACTA	488	Bas: 23<br>GJ: 443<br>XI: 7<br>admix: 15
	Hap3	CGCG-CA	298	Aus: 30<br>GJ: 2<br>XI: 259<br>admix: 6<br>na: 1
	Hap4	CG---CA	279	Aus: 20<br>GJ: 2<br>XI: 252<br>admix: 5
	Hap5	AGCA-TA	191	Bas: 14<br>GJ: 164<br>XI: 3<br>admix: 10
	Hap6	CG--TCA	87	Aus: 6<br>GJ: 1<br>XI: 76<br>admix: 4
				Bas: 8

	Hap7	AG---TA	80	GJ: 64<br>XI: 3<br>admix: 5
CT-15	Hap8	CGCGTCM	71	Aus: 5<br>XI: 65<br>admix: 1
	Hap9	CKCGTCA	55	Aus: 2<br>GJ: 1<br>XI: 51<br>admix: 1
	Hap10	AGCACTM	52	GJ: 52
	Hap11	AGCGCCA	43	Aus: 17<br>Bas: 7<br>XI: 18<br>admix: 1
	Hap12	AGCG-CA	28	Aus: 14<br>Bas: 3<br>XI: 9<br>admix: 2
	Hap13	AG--CTA	25	Bas: 1<br>GJ: 24
	Hap14	CKCG-CA	25	Aus: 2<br>GJ: 1<br>XI: 22
	Hap15	CK---CA	23	Aus: 3<br>XI: 20
	Hap16	AG---CA	22	Aus: 10<br>Bas: 4<br>XI: 6<br>admix: 2
	Hap17	AKCACTA	21	Bas: 2<br>GJ: 19
	Hap18	CGTGTCA	20	XI: 20
	Hap1	CC	1940	Aus: 148<br>Bas: 19<br>GJ: 15<br>XI: 1714<br>admix: 43<br>na: 1
CT-16	Hap2	AC	921	Bas: 54<br>GJ: 821<br>XI: 14<br>admix: 32
	Hap3	CG	86	Aus: 49<br>XI: 36<br>admix: 1
	Hap4	MC	58	Aus: 1<br>Bas: 3<br>GJ: 14<br>XI: 16<br>admix: 24
	Hap1	CCCCGG	1388	Aus: 101<br>Bas: 14<br>GJ: 10<br>XI: 1234<br>admix: 28

				na: 1
CT-17	Hap2	TCTTCG	716	Bas: 34<br>GJ: 645<br>XI: 12<br>admix: 25
	Hap3	CCCC--	272	Aus: 25<br>Bas: 3<br>GJ: 1<br>XI: 237<br>admix: 6
	Hap4	TCTT--	111	Bas: 12<br>GJ: 91<br>XI: 2<br>admix: 6
	Hap5	CCCC-G	97	Aus: 11<br>Bas: 3<br>GJ: 1<br>XI: 79<br>admix: 3
	Hap6	CCCCG-	88	Aus: 7<br>GJ: 3<br>XI: 74<br>admix: 4
	Hap7	CTCCGG	60	Aus: 34<br>XI: 24<br>admix: 2
	Hap8	CCCCGA	49	XI: 49
	Hap9	TCTT-G	37	Bas: 2<br>GJ: 34<br>admix: 1
	Hap10	TCTTC-	29	GJ: 29
	Hap11	CTCC--	15	Aus: 7<br>XI: 8
CT-18	Hap1	AGGGCCGGATG	1899	Aus: 145<br>Bas: 20<br>GJ: 15<br>XI: 1681<br>admix: 37<br>na: 1
	Hap2	GGGACCGGAGG	659	Bas: 48<br>GJ: 569<br>XI: 14<br>admix: 28
	Hap3	GGGRYYGKAGG	109	GJ: 106<br>admix: 3
	Hap4	AGAGCCGGGTA	85	Aus: 48<br>XI: 35<br>admix: 2
	Hap5	GGGACCAGAGG	80	GJ: 79<br>admix: 1
	Hap6	GAGACCGGAGG	36	GJ: 36
	Hap7	RGGRCCGGAKG	26	GJ: 3<br>XI: 10<br>admix: 13
				Aus: 141

CT-19	Hap1	CACCCCCGT	1861	Bas: 6<br>GJ: 15<br>XI: 1665<br>admix: 33<br>na: 1
	Hap2	CGCCCTCAC	661	Bas: 2<br>GJ: 627<br>XI: 12<br>admix: 20
	Hap3	CGCTCTCAC	220	Bas: 47<br>GJ: 160<br>XI: 1<br>admix: 12
	Hap4	TATCTCTGT	81	Aus: 46<br>XI: 33<br>admix: 2
	Hap5	CGCCCCCGC	20	Bas: 13<br>XI: 2<br>admix: 5
	Hap6	CRCCCYCRY	20	GJ: 3<br>XI: 5<br>admix: 12
CT-20	Hap1	TCATCACCGA	926	Aus: 1<br>Bas: 57<br>GJ: 1<br>XI: 843<br>admix: 24
	Hap2	TCCCCGCTCG	880	Aus: 1<br>Bas: 9<br>GJ: 811<br>XI: 33<br>admix: 26
	Hap3	TTCCAGATCG	764	Aus: 36<br>Bas: 3<br>GJ: 8<br>XI: 699<br>admix: 17<br>na: 1
	Hap4	CCCCCGGTCG	226	Aus: 156<br>Bas: 1<br>XI: 64<br>admix: 5
	Hap5	TYMYMRMYSR	19	XI: 17<br>admix: 2
	Hap6	TYCCMGMTCG	17	GJ: 4<br>XI: 6<br>admix: 7
	Hap1	GGGG	1493	Aus: 190<br>Bas: 32<br>GJ: 1<br>XI: 1231<br>admix: 38<br>na: 1
	Hap2	GCGG	944	Aus: 3<br>Bas: 40<br>GJ: 832<br>XI: 34<br>admix: 35

CT-21	Hap3	GGGT	348	XI: 344<br>admix: 4
	Hap4	AGAG	64	Aus: 6<br>XI: 57<br>admix: 1
	Hap5	GSGG	39	Bas: 1<br>GJ: 10<br>XI: 18<br>admix: 10
	Hap6	GGGK	28	XI: 26<br>admix: 2
	Hap7	-GGG	18	XI: 17<br>admix: 1
	Hap1	T	1704	Aus: 151<br>GJ: 5<br>XI: 1516<br>admix: 32
CT-22	Hap2	C	1227	Aus: 45<br>Bas: 73<br>GJ: 834<br>XI: 229<br>admix: 45<br>na: 1
	Hap3	Y	80	Aus: 4<br>Bas: 2<br>GJ: 12<br>XI: 37<br>admix: 25
	Hap1	AGCCTTT	1323	Aus: 30<br>GJ: 1<br>XI: 1274<br>admix: 18
	Hap2	GGCACCT	694	Aus: 1<br>Bas: 47<br>GJ: 613<br>XI: 14<br>admix: 19
	Hap3	GTTCCTA	148	Aus: 102<br>Bas: 1<br>GJ: 1<br>XI: 38<br>admix: 5<br>na: 1
	Hap4	-GCACCT	123	Bas: 10<br>GJ: 102<br>XI: 4<br>admix: 7
	Hap5	GGCCCTT	120	Aus: 6<br>GJ: 2<br>XI: 107<br>admix: 5
	Hap6	-GCCTTT	76	Aus: 1<br>XI: 73<br>admix: 2
CT-23	Hap7	A-CCTTT	48	XI: 48
	Hap8	-TTCCTA	31	Aus: 23<br>XI: 6

				admix: 2
	Hap9	G-CACCT	24	Bas: 1<br>GJ: 21<br>XI: 1<br>admix: 1
	Hap10	-GCCCTT	24	XI: 22<br>admix: 2
	Hap11	--CACCT	23	Bas: 3<br>GJ: 18<br>XI: 2
	Hap12	A---TTT	19	Aus: 1<br>XI: 17<br>admix: 1
	Hap13	--CCTTT	16	XI: 16
	Hap14	----CCT	16	Bas: 1<br>GJ: 14<br>XI: 1
	Hap15	GG--CCT	15	Bas: 1<br>GJ: 11<br>XI: 1<br>admix: 2
CT-24	Hap1	TTGCG	1168	Aus: 33<br>Bas: 1<br>GJ: 1<br>XI: 1113<br>admix: 20
	Hap2	CCGCG	1165	Aus: 161<br>Bas: 69<br>GJ: 812<br>XI: 76<br>admix: 46<br>na: 1
	Hap3	TTTCG	239	XI: 237<br>admix: 2
	Hap4	TTGTG	165	XI: 162<br>admix: 3
	Hap5	TTGCA	104	XI: 103<br>admix: 1
	Hap6	YYGCG	27	GJ: 7<br>XI: 5<br>admix: 15
	Hap7	TTKCG	19	XI: 18<br>admix: 1
	Hap8	YCGCG	15	Aus: 2<br>Bas: 1<br>GJ: 9<br>XI: 1<br>admix: 2
	Hap1	TGCC	1058	Aus: 23<br>Bas: 1<br>GJ: 1<br>XI: 1012<br>admix: 21
	Hap2	TTCC	674	Aus: 7<br>Bas: 40<br>GJ: 583<br>XI: 21

				admix: 23
	Hap3	TG-C	402	Aus: 10<br>Bas: 1<br>XI: 385<br>admix: 6
	Hap4	TT-C	204	Aus: 2<br>Bas: 22<br>GJ: 164<br>XI: 3<br>admix: 13
	Hap5	CTCC	123	Aus: 88<br>Bas: 1<br>GJ: 1<br>XI: 27<br>admix: 5<br>na: 1
CT-25	Hap6	TGMC	91	XI: 89<br>admix: 2
	Hap7	T--C	66	Aus: 2<br>Bas: 3<br>GJ: 12<br>XI: 49
	Hap8	CT-C	56	Aus: 40<br>XI: 14<br>admix: 2
	Hap9	TTMC	56	Bas: 2<br>GJ: 51<br>XI: 1<br>admix: 2
	Hap10	TGCY	47	XI: 47
	Hap11	T-CC	40	Bas: 1<br>GJ: 12<br>XI: 26<br>admix: 1
	Hap12	TKCC	32	Bas: 2<br>GJ: 10<br>XI: 9<br>admix: 11
	Hap13	TG-Y	20	XI: 20
	Hap14	TGAC	17	XI: 16<br>admix: 1
	Hap1	TAGACAGGATTCTCCCTGTAC<br>AGCCGGTGCCCACCATGCATG<br>TTGGCTATTTCCAGCACAAAG<br>TATTGACATGCGGAGCTTGAC<br>CATCCACCGGGTGGCGTGGCC<br>TTCTAAATACCTAAACTTTTCC<br>[illegible]	704	Aus: 2<br>Bas: 52<br>GJ: 622<br>XI: 6<br>admix: 22
	Hap2	GAGCCGGGATGCTCCCTGTAC<br>AGCCGGTGCCCATAATGCATG<br>TTGGGTATTTCCAGCACGAAG<br>TATTGACACGCGGAGCTTGAC<br>CATTCACCGGGTGACGTGGCC<br>TTCTGAATACCTAAACTTTTCT<br>GTTGAGGCCCTCCGCG	175	Aus: 95<br>Bas: 4<br>GJ: 2<br>XI: 62<br>admix: 11<br>na: 1
		GAGCCRGGATGCTCCCTGTAC<br>AGCCGGTGCCCATAATGCATG<br>[illegible]		Aus: 68<br>XI: 32

	Hap3	TTGGGTATTTCCAGCACGAAG<br>TATTGACACGCGGAGCTTGAC<br>CATTCACCGGGTGACGTGGCC<br>TTCTGAATACCTAAACTTTTCT<br>[illegible]	104	admix: 4
CT-26	Hap4	TAGACAGGATTCTCCCTGTAC<br>AGCCGGTGCCCACCATGCATG<br>TTGGCTATTTCCAGCACAAAG<br>TATTGACATGCGGAGCTTGAC<br>CATCCACCGGGTGGCGTGGCC<br>TTCTAAATACCTAAATTTTTCC<br>[illegible]	26	GJ: 26
	Hap5	TAGACAGGATTCTCCCTGTAC<br>AGCCGGTGCCCACCATGCATG<br>TTGGCTATTTCCAGCACAAAG<br>TATTGACATGCGGAGCTTGAC<br>CATCCACCGGGTAGCGTGGCC<br>TTCTAAATACCTAAACTTTTCC<br>[illegible]	24	GJ: 24
	Hap6	TAGCAGGATTCTCCCTGTACA<br>GCCGGTGCCCACCATGCATGT<br>TGGCTATTTCCAGCACAAAGT<br>ATTGACATGCGGAGCTTGACC<br>ATCCACCGGGTGGCGTGGCCT<br>TCTAAATACCTAAACTTTTCC<br>[illegible]	21	Bas: 3<br>GJ: 16<br><br>admix: 2
	Hap7	GGGACGGAATGTTCCCAGTAA<br>AGCCGGTGCCCACCATGCATG<br>TTGGGTATTTCCAGCAGGAAG<br>TATTGACACGCGGAGCTTGAC<br>CATCCATCGAGTGGC-------------	19	GJ: 19
	Hap1	CCTAGCCCCCC	1664	Aus: 1<br>Bas: 1<br>GJ: 25<br>XI: 1611<br>admix: 26
	Hap2	CCTAACCACCC	820	Aus: 1<br>Bas: 47<br>GJ: 745<br>XI: 5<br>admix: 22
CT-27	Hap3	TCTAGGTCCTC	344	Aus: 192<br>Bas: 21<br>GJ: 1<br>XI: 108<br>admix: 21<br>na: 1
	Hap4	CTCTGCCCACT	53	GJ: 51<br>XI: 1<br>admix: 1
	Hap5	CCTARCCMCCC	24	GJ: 7<br>XI: 6<br>admix: 11
	Hap6	YCTAGSYCCYC	16	Aus: 1<br>Bas: 1<br>XI: 11<br>admix: 3
