# Supplementary material for: High‐throughput selective sweep SNP‐guided cloning of cold‐tolerance genes in rice
Source: Plant Biotechnol J. 2024 Mar 7;22(8):2104–6. doi: 10.1111/pbi.14329 (PMC11258967; doi:10.1111/pbi.14329)
Supplement: Supplementary file 5 — Table S5 The genomic mutations in the homozygous mutant lines for the candidate genes. [file PBI-22-2104-s002.pdf]

Supporting Table S5. The genomic mutations in the homozygous mutant lines for the candidate genes

Lines		Sequence	CDS	Mutation type
CT-1	Ref:	GGCGACGGCGTGCCGCTCGGCGGCGGCCGGAT-CAGGTTCGAGTTCTTGG	477aa	
#30	Allele 1:	CTCCTCCA·············· (-25bp) ···········AGGTTCGAGTTCTTGG	93aa	Hom
	Allele 2:	CTCCTCCA·············· (-25bp) ···········AGGTTCGAGTTCTTGG	93aa	
#31	Allele 1:	CCGGATTCAGGTTCGAGTTCTTGG	475aa	Hom
	Allele 2:	CCGGATTCAGGTTCGAGTTCTTGG	475aa	
CT-2	Ref:	CCTT-CCTGCAGTGCAAGCATCCA	176aa	
#5	Allele 1:	CC---------------GCATCCA	104aa	Hom
	Allele 2:	CC---------------GCATCCA	104aa	
#6	Allele 1:	CCTT-----------AAGCATCCA	174aa	Bia
	Allele 2:	CCTTACATGGA(-17bp)	101aa	
#21	Allele 1:	CC-----------------ATCCA	80aa	Bia
	Allele 2:	CCTT-CC-----TGCAAGCATCCA	175aa	
CT-3	Ref:	TGATCAGGTGCTCAACCATGGGG	342aa	
#9	Allele 1:	TGATCAGGTGCTCAAC-ATGGGG	74aa	Hom
	Allele 2:	TGATCAGGTGCTCAAC-ATGGGG	74aa	
#17	Allele 1:	TGATCAGGTGCTCAAC-ATGGGG	74aa	Bia
	Allele 2:	TGATCAGGTG-----------GG	134aa	
CT-4	Ref:	CCGTGCT-29TTGTTCGCGTCGAC	237aa	
#34	Allele 1:	CCGTGCTTACTTGTTCGCGTCGAC	119aa	Hom
	Allele 2:	CCGTGCTTACTTGTTCGCGTCGAC	119aa	
#35	Allele 1:	CCGTGC--ACTTGTTCGCGTCGAC	143aa	Hom
	Allele 2:	CCGTGC--ACTTGTTCGCGTCGAC	143aa	
CT-5	Ref:	AAGGCATTGGTCTGGTAAGCGGG	1059aa	
#2	Allele 1:	GCC(-55bp)TTGGGGGC	43aa	Hom
	Allele 2:	GCC(-55bp)TTGGGGGC	43aa	
#4	Allele 1:	AAG-CATTGGTCTGGTAAGCGGG	52aa	Bia
	Allele 2:	AAG-----GGTCTGGTAAGCGGG	76aa	
#34	Allele 1:	AAG-CATTGGTCTGGTAAGCGGG	52aa	Hom
	Allele 2:	AAG-CATTGGTCTGGTAAGCGGG	52aa	
CT-6	Ref:	CCATCCCAGTCCAGCACGCTAC-TCCAGG	349aa	
#28	Allele 1:	CCATCCCAGTCCAGCACGCTACATCCAGG	147aa	Bia
	Allele 2:	CC (-58bp)	161aa	
#35	Allele 1:	CCAG--------------TCCAGG	176aa	Hom
	Allele 2:	CCAG--------------TCCAGG	176aa	
#42	Allele 1:	CCATCCCAGTCCAGCACGCTACTTCCAGG	147aa	Bia
	Allele 2:	CC (-58bp)	161aa	
CT-7	Ref:	GACCGGCTGCAAAGGCCCTTCGG	127aa	
#12	Allele 1:	GACCGGCTGCAAAGGCC-TTCGG	54aa	Bia
	Allele 2:	GACCGGCT(-22bp)	47aa	
#23	Allele 1:	GACCGGCTGCAAAGGCC-TTCGG	54aa	Hom
	Allele 2:	GACCGGCTGCAAAGGCC-TTCGG	54aa	
#39	Allele 1:	GACCGG-----------CTTCGG	36aa	Bia
	Allele 2:	GACCGGCTGCAAAG--CCTTCGG	39aa	
CT-8	Ref:	CCGG--CC-CCTCGTTCGGCTTCGTC	213aa	
#2	Allele 1:	CCGGCCCC-CCTCGTTCGGCTTCGTC	104aa	Hom
	Allele 2:	CCGGCCCC-CCTCGTTCGGCTTCGTC	104aa	
#4	Allele 1:	CCGG--CC-C-TCGTTCGGCTTCGTC	103aa	Hom
	Allele 2:	CCGG--CC-C-TCGTTCGGCTTCGTC	103aa	
#15	Allele 1:	CCGG--CCTCCTCGTTCGGCTTCGTC	143aa	Bia
	Allele 2:	CCGG--CC-----GTTCGGCTTCGTC	102aa	
CT-9	Ref:	CCCAGC-CAAGTGAGTACCCGTCG	173aa	
#3	Allele 1:	CCCAGC-CA-----GTACCCGTCG	-	Bia
	Allele 2:	C------------AGTACGCGTCG	170aa	
#35	Allele 1:	CCCAGCTCAAGTGAGTACCCGTCG	-	Bia
	Allele 2:	CCCAGCACAAGTGAGTACCCGTCG	-	
CT-10	Ref:	CCCAAGAGCCACCGCGCCAAGAG	111aa	
#3	Allele 1:	(-10bp)CACCGCGCCAAGAG	38aa	Hom
	Allele 2:	(-10bp)CACCGCGCCAAGAG	38aa	
#25	Allele 1:	(-16bp)GCGCCAAGAG	36aa	Hom
	Allele 2:	(-16bp)GCGCCAAGAG	36aa	
#28	Allele 1:	CCCAAG-GCCACCGCGCCAAGAG	41aa	Hom
	Allele 2:	CCCAAG-GCCACCGCGCCAAGAG	41aa	
CT-11	Ref:	CCGCCTCGGGCTGCATCTTCCTC	166aa	
#1	Allele 1:	CCGCCT-GGGCTGCATCTTCCTC	113aa	Hom
	Allele 2:	CCGCCT-GGGCTGCATCTTCCTC	113aa	
#10	Allele 1:	CCGCCT--GGCTGCATCTTCCTC	-	Hom
	Allele 2:	CCGCCT--GGCTGCATCTTCCTC	-	
CT-12	Ref:	TGGCGAATGCCCAGCAGTTCTGG	1369aa	
#9	Allele 1:	TGGCGAATGCCCAGCAG(-19bp)	56aa	Bia
	Allele 2:	TGGCGAATGCCCAGC--TTCTGG	29aa	
#26	Allele 1:	TGGCGAATGCCCAGCAGT-CTGG	62aa	Hom
	Allele 2:	TGGCGAATGCCCAGCAGT-CTGG	62aa	
CT-13	Ref:	GATCATCAAACCGCTCC-AGCGGG	134aa	
#8	Allele 1:	GATCATCAAACCGCTCCTAGCGGG	62aa	Bia
	Allele 2:	GATCATCAAACCG---------GG	91aa	
#11	Allele 1:	GATCATCAAACCGCTC-----GGG	83aa	Bia
	Allele 2:	GATCATCAAA---------GCGGG	91aa	

Supporting Table S4 Continued.

Lines	Sequence	CDS	Mutation type
CT-14	Ref: AGTCTTGCAATGATGTC-AAGAGG	157aa	
#14	Allele 1: AGTCTTGCAATGATGTCAAAGAGG	37aa	Hom
	Allele 2: AGTCTTGCAATGATGTCAAAGAGG	37aa	
#27	Allele 1: AGTCTTGCAATGATGTCCAAGAGG	37aa	Bia
	Allele 2: AGTCTTGCAATGATGTCAAAGAGG	37aa	
CT-15	Ref: GCGTCCAAGGCGTACAT-CGACGG	264aa	
#19	Allele 1: GCGTCCAAGGCGTACATTCGACGG	239aa	Hom
	Allele 2: GCGTCCAAGGCGTACATTCGACGG	239aa	
#20	Allele 1: GCGTCCAAGGCGTACATCCGACGG	239aa	Hom
	Allele 2: GCGTCCAAGGCGTACATCCGACGG	239aa	
CT-16	Ref: AGGGAGAAGGGCCTCAGAGCCGG	272aa	
#8	Allele 1: AGGGAGAAGGGCC--------GG	175aa	Bia
	Allele 2: AGGGAGAAGGGCCTC--AGCCGG	177aa	
#20	Allele 1: AGGGAGAAGGGC-----AGCCGG	176aa	Bia
	Allele 2: AGGGAGAAGGGCCAGCCGAAA(-4bp)	179aa	
#41	Allele 1: AGGGAGAAGGGC-----AGCCGG	176aa	Bia
	Allele 2: AGGGAGAAGGGCC----AGCCGG	133aa	
CT-17	Ref: CCGTCCCTCGACTTCGGCGACCC	561aa	
#12	Allele 1: CCGTCCA---ACTTCGGCGACCC	560aa	Bia
	Allele 2: CCGTCCA---ACTTCGGCGACCC	560aa	
#13	Allele 1: C---------ACTTCGGCGACCC	558aa	Hom
	Allele 2: C---------ACTTCGGCGACCC	558aa	
CT-18	Ref: AAAGAGACCAAT-GGGTAGGAAGG	320aa	
#6	Allele 1: A(-52bp)	209aa	Hom
	Allele 2: A(-52bp)	209aa	
#38	Allele 1: AAAGAGACCAATAGGGTAGAGAGG	206aa	Bia
	Allele 2: AAAGAGACCA-TAGGG-AG--AAG	204aa	
CT-19	Ref: TGACAAGGCGGCTTCGACTAGGGGCGACAAGG	120aa	
#44	Allele 1: TGACAA (-16bp)	26aa	Hom
	Allele 2: TGACAA (-16bp)	26aa	
#46	Allele 1: (-41bp)AGG	20aa	Hom
	Allele 2: (-41bp)AGG	20aa	
CT-20	Ref: GAAAACGTCGTAGCTGC-TGACGG	122aa	
#9	Allele 1: GAAAACGTCGTAGCTGCATGACGG	79aa	Bia
	Allele 2: GAAAACGTCGTAGCTGC(-32bp)	83aa	
#27	Allele 1: GAAAACGTCGTAGCTGCTTGACGG	79aa	Hom
	Allele 2: GAAAACGTCGTAGCTGCTTGACGG	79aa	
CT-21	Ref: GGCTGCTGCAATTCAGA-CGCAGG	99aa	
#2	Allele 1: GGCTGCTGCAATTCAGATCGCAGG	63aa	Bia
	Allele 2: GGCTGCTGCAATTCAGAACGCAGG	63aa	
#10	Allele 1: GGCTGCTGCAATTCAGATCGCAGG	63aa	Hom
	Allele 2: GGCTGCTGCAATTCAGATCGCAGG	63aa	
#15	Allele 1: GGCTGCTGCAATTCAGAACGCAGG	63aa	Hom
	Allele 2: GGCTGCTGCAATTCAGAACGCAGG	63aa	
CT-22	Ref: GTCAAAATTCACTCGCTTCAAGG	181aa	
#3	Allele 1: GTCAAAATTCACTCGCT-CAAGG	37aa	Hom
	Allele 2: GTCAAAATTCACTCGCT-CAAGG	37aa	
CT-24	Ref: CTATCAAACCCTTGGGA-GATAGG	245aa	
#3	Allele 1: CTATCAAACCCTTGGGAAGATAGG	60aa	Bia
	Allele 2: (-80bp) ATAGG	236aa	
#9	Allele 1: CTATCAAACCCTTGGGA---TAGG	59aa	Bia
	Allele 2: CTATCAAACCCTTGGGAAGATAGG	60aa	
#11	Allele 1: CTATCAAACCCTTGGGAAGATAGG	60aa	Hom
	Allele 2: CTATCAAACCCTTGGGAAGATAGG	60aa	
CT-25	Ref: TGGTCTTTGCAGGTGCT-20TCCGG	232aa	
#3	Allele 1: TGGTCTTTGCAGGTGCT-20-CCGG	69aa	Hom
	Allele 2: TGGTCTTTGCAGGTGCT-20-CCGG	69aa	
#12	Allele 1: (-29bp)GG	107aa	Hom
	Allele 2: (-29bp)GG	107aa	
CT-26	Ref: CCGCGGATGCGATCGTTGTCAGC	681aa	
#4	Allele 1: CCGCGG--GCGATCGTTGTCAGC	201aa	Hom
	Allele 2: CCGCGG--GCGATCGTTGTCAGC	201aa	
#9	Allele 1: CCGCGGAG(-16bp)	206aa	Bia
	Allele 2: (-33bp)TCGTTGTCAGC	670aa	
CT-27	Ref: AGCGACAGGGATGACGA-TATTGG	495aa	
#7	Allele 1: AGCGACAGG------GA-TATTGG	493aa	Bia
	Allele 2: AGCGACAGGGATGA---------G	53aa	
#10	Allele 1: AGCGACAGGGATGAC------TGG	54aa	Hom
	Allele 2: AGCGACAGGGATGAC------TGG	54aa	
#14	Allele 1: AGCGACAGGGATGACGAATATTGG	56aa	Hom
	Allele 2: AGCGACAGGGATGACGAATATTGG	56aa	
