# Supplementary material for: High‐throughput selective sweep SNP‐guided cloning of cold‐tolerance genes in rice
Source: Plant Biotechnol J. 2024 Mar 7;22(8):2104–6. doi: 10.1111/pbi.14329 (PMC11258967; doi:10.1111/pbi.14329)
Supplement: Supplementary file 6 — Table S6 Primers for introducing the single guide RNAs (sgRNAs) for the CRISPR/Cas9 gene editing of the candidate genes. Table S7 Primers used for the qRT‐PCR analysis of the cold‐tolerance genes. [file PBI-22-2104-s003.pdf]

Supporting Table S6. Primers for introducing the single guide RNAs (sgRNAs) for the CRISPR/Cas9 gene editing of the candidate genes

Candidate	Primer	sgRNA sequence (5' to 3')
CT-1	U6aF	GATCAGGTTCGAGTTCTTGGcggcagccaagccagca
	U6aR	CCAAGAACTCGAACCTGATCgttttagagctagaaat
CT-2	U6aF	TCCTGCAGTGCAAGCATCCAcggcagccaagccagca
	U6aR	TGGATGCTTGCACTGCAGGAgttttagagctagaaat
CT-3	U6aF	TGATCAGGTGCTCAACCATGgttttagagctagaaat
	U6aR	CATGGTTGAGCACCTGATCAcggcagccaagccagca
CT-4	U6aF	TGCTACTTGTTCGCGTCGACgttttagagctagaaat
	U6aR	GTCGACGCGAACAAGTAGCAcggcagccaagccagca
CT-5	U6aF	AAGGCATTGGTCTGGTAAGcggcagccaagccagca
	U6aR	CTTACCAGACCAATGCCTTgttttagagctagaaat
CT-6	U6aF	CCAGTCCAGCACGCTACTCCgttttagagctagaaat
	U6aR	GGAGTAGCGTGCTGGACTGGcggcagccaagccagca
CT-7	U6aF	GACCGGCTGCAAAGGCCCTTgttttagagctagaaat
	U6aR	AAGGGCCTTTGCAGCCGGTCcggcagccaagccagca
CT-8	U6aF	GCCCCTCGTTCGGCTTCGTcggcagccaagccagca
	U6aR	ACGAAGCCGAACGAGGGGCgttttagagctagaaat
CT-9	U6aF	AGCCAAGTGAGTACCCGTCGcggcagccaagccagca
	U6aR	CGACGGGTACTCACTTGGCTgttttagagctagaaat
CT-10	U6aF	AAGAGCCACCGCGCCAAGAGcggcagccaagccagca
	U6aR	CTCTTGGCGCGGTGGCTCTTgttttagagctagaaat
CT-11	U6aF	CCTCGGGCTGCATCTTCCTcggcagccaagccagca
	U6aR	AGGAAGATGCAGCCCGAGGgttttagagctagaaat
CT-12	U6aF	TGGCGAATGCCCAGCAGTTCgttttagagctagaaat
	U6aR	GAACTGCTGGGCATTCGCCAcggcagccaagccagca
CT-13	U6aF	ATCATCAAACCGCTCCAGCgttttagagctagaaat
	U6aR	GCTGGAGCGGTTTGATGATcggcagccaagccagca

CT-14	U6aF	AGTCTTGCAATGATGTCAAGgttttagagctagaaat
	U6aR	CTTGACATCATTGCAAGACTcggcagccaagccagca
CT-15	U6aF	CGTCCAAGGCGTACATCGAgttttagagctagaaat
	U6aR	TCGATGTACGCCTTGGACGcggcagccaagccagca
CT-16	U6aF	AGGGAGAAGGGCCTCAGAGCgttttagagctagaaat
	U6aR	GCTCTGAGGCCCTTCTCCCTcggcagccaagccagca
CT-17	U6aF	TCCCTCGACTTCGGCGACCcggcagccaagccagca
	U6aR	GGTCGCCGAAGTCGAGGGAgttttagagctagaaat
CT-18	U6aF	TCCTACCCATTGGTCTCTTTcggcagccaagccagca
	U6aR	AAAGAGACCAATGGGTAGGAgttttagagctagaaat
CT-19	U6aF	TGACAAGGCGGCTTCGACTAgttttagagctagaaat
	U6aR	TAGTCGAAGCCGCCTTGTCAcggcagccaagccagca
CT-20	U6aF	AAAACGTCGTAGCTGCTGAgttttagagctagaaat
	U6aR	TCAGCAGCTACGACGTTTTcggcagccaagccagca
CT-21	U6aF	GCTGCTGCAATTCAGACGCgttttagagctagaaat
	U6aR	GCTGCTGCAATTCAGACGCgttttagagctagaaat
CT-22	U6aF	TCAAAATTCACTCGCTTCAgttttagagctagaaat
	U6aR	TGAAGCGAGTGAATTTTGAcggcagccaagccagca
CT-23	U6aF	TATCTGCAAGGAATTGGTGgttttagagctagaaat
	U6aR	CACCAATTCCTTGCAGATAcggcagccaagccagca
CT-24	U6aF	TATCAAACCCTTGGTTCGATgttttagagctagaaat
	U6aR	ATCGAACCAAGGGTTTGATAcggcagccaagccagca
CT-25	U6aF	TGGTCTTTGCAGGTGCTTTCgttttagagctagaaat
	U6aR	GAAAGCACCTGCAAAGACCAcggcagccaagccagca
CT-26	U6aF	CGGATGCGATCGTTGTCAGcggcagccaagccagca
	U6aR	CTGACAACGATCGCATCCGgttttagagctagaaat
CT-27	U6aF	AGCGACAGGGATGACGATATgttttagagctagaaat
	U6aR	ATATCGTCATCCCTGTCGCTcggcagccaagccagca

Supporting Table S7. Primers used for the qRT-PCR analysis of the cold-tolerance genes

Gene	Primer		Primer sequence (5' to 3') for qRT-PCR
Reference	*Actin1*	qRTF	GCATCTCTCAGCACATTCCA
		qRTR	ACCACAGGTAGCAATAGGTA
Cold-tolerant	CT-5	qRTF	GCAGTTTGCTTGGTGAGCTG
		qRTR	GATGCTGCTCGAACAACTGC
	CT-9	qRTF	CATCTACGACATGTCGTCC
		qRTR	CGACTTGTATGGCTTCTCC
	CT-10	qRTF	GCTTGTTCTCCATGGGACCA
		qRTR	ACTCAAGCGAAGTTTCGCCG
	CT-17	qRTF	GGGTGCAAAGCTCTTTCTTGG
		qRTR	GACCTCTGAACAGAGCTCCT
	CT-19	qRTF	TCGCCGGTCATCCTCTTGTT
		qRTR	GGATTTGAGGCTACATCCTC
	CT-24	qRTF	AGGACCATTGGTTGAGGATGG
		qRTR	CCATTACGTCGGATACCCTG
	CT-26	qRTF	GCATGCAGCTGATGTGATCG
		qRTR	CAGCTGTGCTTAGCTTCCAG
